# Lung emphysema and impaired macrophage elastase clearance in mucolipin 3 deficient mice

**DOI:** 10.1038/s41467-021-27860-x

**Published:** 2022-01-14

**Authors:** Barbara Spix, Elisabeth S. Butz, Cheng-Chang Chen, Anna Scotto Rosato, Rachel Tang, Aicha Jeridi, Veronika Kudrina, Eva Plesch, Philipp Wartenberg, Elisabeth Arlt, Daria Briukhovetska, Meshal Ansari, Gizem Günes Günsel, Thomas M. Conlon, Amanda Wyatt, Sandra Wetzel, Daniel Teupser, Lesca M. Holdt, Fabien Ectors, Ingrid Boekhoff, Ulrich Boehm, Jaime García-Añoveros, Paul Saftig, Martin Giera, Sebastian Kobold, Herbert B. Schiller, Susanna Zierler, Thomas Gudermann, Christian Wahl-Schott, Franz Bracher, Ali Önder Yildirim, Martin Biel, Christian Grimm

**Affiliations:** 1 Walther Straub Institute of Pharmacology and Toxicology, Faculty of Medicine, Ludwig-Maximilians-University, Munich, Germany; 2 Department of Pharmacy, Ludwig-Maximilians-University, Munich, Germany; 3 Institute for Neurophysiology, Hannover Medical School, Hannover, Germany; 4 Department of Clinical Laboratory Sciences and Medical Biotechnology, College of Medicine, National Taiwan University, Taipei, Taiwan; 5 Comprehensive Pneumology Center, Institute of Lung Biology and Disease, Helmholtz Zentrum München, Member of the German Center for Lung Research (DZL), Munich, Germany; 6 Saarland University, Center for Molecular Signaling (PZMS), Experimental Pharmacology, Homburg, Germany; 7 Division of Clinical Pharmacology, Department of Medicine IV, University Hospital Munich, Munich, Germany; 8 Institute of Biochemistry, Christian-Albrechts-University Kiel, Kiel, Germany; 9 Institute of Laboratory Medicine, University Hospital Munich, Munich, Germany; 10 FARAH Mammalian Transgenics Platform, Liège University, Liège, Belgium; 11 Departments of Anesthesiology, Physiology and Neurology, Northwestern University, Feinberg School of Medicine, Chicago, IL USA; 12 Center for Proteomics and Metabolomics, Leiden University Medical Center, 2333ZA Leiden, The Netherlands; 13 German Center for Translational Cancer Research (DKTK), partner site Munich, Munich, Germany; 14 Institute of Pharmacology, Johannes-Keppler-University, Linz, Australia; 15 German Center of Lung Research (DZL), Munich, Germany

**Keywords:** Endocytosis, Respiration, Pharmacology, Lysosomes, Mechanisms of disease

## Abstract

Lung emphysema and chronic bronchitis are the two most common causes of chronic obstructive pulmonary disease. Excess macrophage elastase MMP-12, which is predominantly secreted from alveolar macrophages, is known to mediate the development of lung injury and emphysema. Here, we discovered the endolysosomal cation channel mucolipin 3 (TRPML3) as a regulator of MMP-12 reuptake from broncho-alveolar fluid, driving in two independently generated *Trpml3*^*−/−*^ mouse models enlarged lung injury, which is further exacerbated after elastase or tobacco smoke treatment. Mechanistically, using a *Trpml3*^*IRES-Cre/eR26-*τ*GFP*^ reporter mouse model, transcriptomics, and endolysosomal patch-clamp experiments, we show that in the lung TRPML3 is almost exclusively expressed in alveolar macrophages, where its loss leads to defects in early endosomal trafficking and endocytosis of MMP-12. Our findings suggest that TRPML3 represents a key regulator of MMP-12 clearance by alveolar macrophages and may serve as therapeutic target for emphysema and chronic obstructive pulmonary disease.

## Abstract (web summary)

Excess macrophage elastase MMP-12 is a major driver of chronic obstructive pulmonary disease. Here the authors show that the endolysosomal ion channel TRPML3 is a regulator of the cellular reuptake of MMP-12, thus neutralizing harmful MMP-12 in the lung.

## Introduction

Chronic obstructive pulmonary disease (COPD) is a global health issue, affecting nearly 300 million people worldwide resulting in the death of about 3 million individuals each year. It develops in response to cigarette smoke or inhalation of environmental and occupational pollutants, such as high levels of dust, e.g., in coal mining, and certain gases, or due to gene defects (e.g., alpha 1-antitrypsin deficiency). The usually observed chronic inflammation in COPD patients is often characterised by increased numbers of macrophages^1–3^, neutrophils^3^, B- and T-lymphocytes^3^ in the airways and lung parenchyma, and there is increasing evidence that these cells play a central role in orchestrating the inflammatory response in COPD^4,5^. Recurrent acute infections by bacterial and/or viral pathogens are also clearly linked with the occurrence of exacerbations of COPD^6^. Alveolar macrophages (AMΦ) are the primary phagocytes of the innate immune system, clearing the air spaces of infectious, toxic, or allergic particles that have evaded the mechanical defenses of the respiratory tract. By secretion of oxygen metabolites, antimicrobial peptides and proteases, and through processes of phagocytosis and intracellular killing, AMΦ can eliminate microbes that are aspirated daily in the normal host. When faced with large numbers of infectious particles or microbes, AMΦ can synthesize and secrete a wide array of inflammatory mediators^7^. The increased secretion of inflammatory mediators sustains the inflammatory process which can lead to tissue damage as well as a range of systemic effects. In patients with emphysema, AMΦ produce an excess of matrix metalloproteinases, in particular MMP-12 (also known as macrophage metalloelastase or macrophage elastase), which contributes to structural changes in the lung^8^. Notably, MMP-12^−/−^ mice do not develop emphysema even after long-term exposure to cigarette smoke^9^. Mucolipins, also called MCOLN or TRPML cation channels are expressed in the endolysosomal system and comprise three members in the mammalian genome. While TRPML1 is ubiquitously expressed, TRPML2 and TRPML3 show more select expression profiles. TRPML1 regulates phagocytosis, endolysosomal trafficking, and lysosomal exocytosis, and TRPML2 has recently been shown to be directly involved in the secretion of chemokines from bone marrow-derived macrophages and to regulate recycling endosomal trafficking^10–14^. Here, we present results from two independently generated and differentially engineered *Trpml3*^−*/*−^ mouse models, revealing lung tissue injury and an emphysema-like phenotype in both *Trpml3*^−/−^ mouse strains, which was further exacerbated after elastase or tobacco smoke treatment. We analysed transcriptomics data, generated a *Trpml3*^IRES-Cre/eR26-τGFP^ reporter mouse model, applied endolysosomal patch-clamp methods, and isoform-selective TRPML agonists to investigate expression and function of TRPML3 in the lung where it was found to be expressed predominantly in AMΦ. Using endolysosomal patch-clamp electrophysiology, we precisely demonstrate where TRPML3 is expressed on a subcellular level in AMΦ. To mechanistically understand how the loss of TRPML3 impacts lung physiology, we performed an in-depth functional analysis of WT versus *Trpml3*^−*/*−^ endolysosomes in AMΦ. Loss of TRPML3 results in endocytosis and early endosomal trafficking defects in AMΦ which endocytose less MMP-12 upon blockade of clathrin-independent endocytosis (macropinoytosis) in a TRPML3-dependent manner, and more MMP-12 when activated with a selective TRPML3 agonist, thus highlighting a new mechanism involved in the regulation of MMP-12 levels in the extracellular matrix of the lungs.

## Results

### *Trpml3*^IRES-Cre/eR26-τGFP^ reporter mouse model reveals selective expression of TRPML3 in AMΦ in the lung

**Fig. 1:**
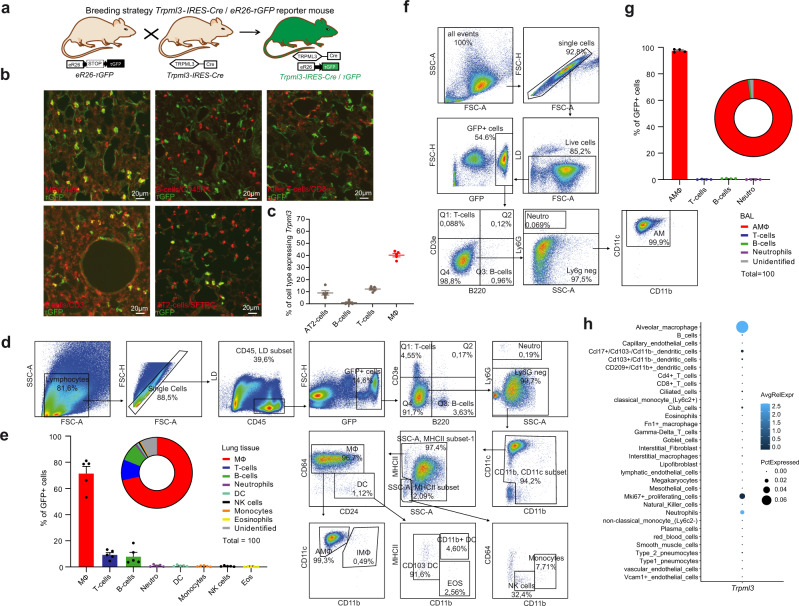
Characterization of TRPML3 expression in the lungs using single-cell transcriptomics and a *Trpml3*^*IRES-Cre/eR26-*τ*GFP*^ reporter mouse model. **a** Cartoon showing the breeding strategy to obtain *Trpml3*^IRES-Cre/eR26-τGFP^ mice. **b** Immunofluorescence images using antibodies against different cell markers (red) in 10 µm lung cryosections from transcardially perfused (4% PFA) *Trpml3*^IRES-Cre/eR26-τGFP^ mice. TRPML3 expression were visually detected in MΦ, T-cells, B-cells, AT2-cells, and Killer T-cells by colocalization analysis with the respective marker. **c** Quantification of data as shown in **b**. Percentage of cell type expressing TRPML3 was determined in five randomly chosen zoom-in sections, each (mean ± SEM). **d**, **f** FACS analysis of lung tissue and BAL of *Trpml3*^IRES-Cre/eR26-τGFP^ mice. Shown in d is the gating strategy used to identify TRPML3 + immune cells in the lungs. Further details are provided in the Methods section. Gating strategy and dot plots revealed TRPML3 being expressed mostly in AMΦ in the lung. **e** Quantitative analysis based on dot plots shown in d. Bar and pie charts show that the highest percentage of GFP + ( = TRPML3+) cells in the lung tissue corresponds to MΦ (71,58%; mean ± SEM, collected from 5 *Trpml3*^IRES-Cre/eR26-τGFP^ mice). **f** Gating strategy used to identify TRPML3+ cells in BAL isolated from *Trpml3*^IRES-Cre/eR26-τGFP^ mice. **g** Quantitative analysis based on dot plots as shown in **f**. Bar and pie charts show that the highest percentage of GFP + ( = TRPML3+) cells in the BAL corresponds to MΦ (97.5%; mean ± SEM, collected from 4 *Trpml3*^IRES-Cre/eR26-τGFP^ mice). **h** Transcriptomics data of single-cell suspensions from whole WT mouse lungs. Dot plot shows the percentage of cells expressing *Mcoln3* using dot size and the average expression level of *Mcoln3* based on the unique molecular identifier (UMI) counts. *Mcoln3* expression was determined in 32 different cell types. Source data are provided as a Source Data file.

The cellular expression of TRPML3 on the whole-tissue level remains largely elusive. To overcome this problem, we generated a GFP reporter mouse model for TRPML3. Briefly, we produced an 8.5 kb targeting construct containing a 2.6 kb 5′ homology arm and a 2.5 kb 3′ homology arm, inserting the IRES-Cre-PGK-Neomycin cassette 5 bp after the stop codon in exon 12 of the *Trpml3* gene. Southern Blot analysis using HpaI and a 32P-labelled 586 bp probe distinguished between the 3.8 kb WT and the correctly targeted 7.3 kb *Trpml3*-IRES-Cre knock-in allele. After blastocyst injection and germline transmission of the *Trpml3*-IRES-Cre allele, heterozygous *Trpml3*-IRES-Cre mice were crossed with FLP deleter mice. *Trpml3*-IRES-Cre neo-, FLP- animals were then crossed to Cre-dependent ROSA26-CAGS-τGFP (eR26-τGFP) fluorescent reporter mice for visualization of gene expression. Thus obtained *Trpml3*^IRES-Cre/eR26-τGFP^ mice (Fig. 1a) were used to analyse the expression pattern of TRPML3 (GFP + cells) in different organs and tissues including lung tissue and bronchoalveolar lavage (BAL). We performed immunofluorescence experiments with antibodies against different cell markers in lung cryosections from transcardially perfused (4% PFA) *Trpml3*^IRES-Cre/eR26-τGFP^ mice, revealing a predominant expression of TRPML3 in macrophages (MΦ) in the lung (Fig. 1b, c). We used FACS (fluorescence-activated cell sorting) to analyze GFP + (TRPML3 +) immune cell populations in the lung in more detail and found the highest percentage of GFP + cells being again MΦ, both in lung tissue and in BAL (AMΦ) (Fig. 1d–g). We complemented these data by transcriptomics analysis of single-cell suspensions from whole WT mouse lungs, which revealed the highest percentage of TRPML3 expression (coded by dot size) in AMΦ, as well as highest average expression levels of TRPML3 (coded by colour grading) in AMΦ (Fig. 1h and Fig. S1)^15^. This was surprising as TRPML3 is largely absent from other macrophage populations, such as peritoneal or bone marrow macrophages (Fig. S2a). Among lung macrophage populations TRPML3 expression is exceptionally high in AMΦ, while being lower in CD11b-positive interstitial lung tissue macrophages (LMΦ) (Fig. S2a). This predominant and high expression of TRPML3 in AMΦ prompted us to assess the lung function in *Trpml3*^−*/*−^ mouse models.

### Loss of TRPML3 affects lung function

**Fig. 2:**
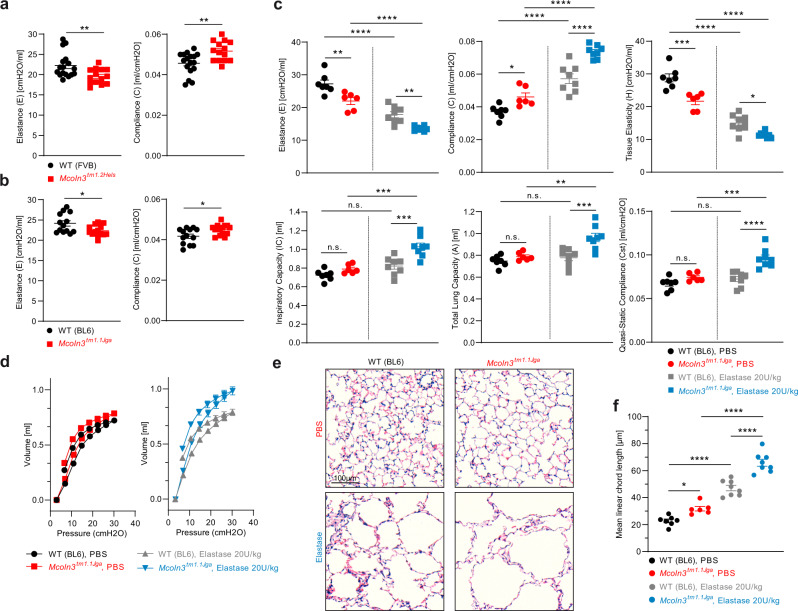
Lung function parameters in WT and *Trpml3*^−*/*−^ mice (*Mcoln3*^*tm1.2Hels*^ and *Mcoln3*^*tm1.1Jga*^). Lung function measurements were performed using the SCIREQs FlexiVent System (see Methods). Different manoeuvres were applied. Single Frequency Forced Oscillation Technique (FOT) allows to study the subject’s response to a sinusoidal waveform, obtaining parameters such as Elastance (E) and Compliance (C). Broadband FOT measures the subject’s response to a signal, including a broad range of frequencies, below and above the subject’s breathing frequency. Outcomes are, e.g., Tissue Elasticity (H). Deep Inflation inflates the lungs to a total lung capacity state. Initial and end volumes are used to calculate Inspiratory Capacity (IC). Pressure-volume (PV) loops capture the quasistatic mechanical properties of the respiratory system such as Quasi-Static Compliance (Cst) and Total Lung Capacity (A). **a**, **b** In two different 4–5 months old *Trpml3*^−*/*−^ mouse models on different background, each (BL6 and FVB), a significant reduction of Elastance (E) of the whole respiratory system was observed, whereas the Compliance (C) was significantly increased (basal, untreated). *p < 0.05, **p < 0.01; Student’s *t* test, unpaired, two-tailed. **c** Differences of E, C, H, Cst, IC, and A in PBS versus elastase-treated 4–5 months old *Trpml3*^−*/*−^ and WT mice. *p < 0.05, **p < 0.01, ***p < 0.001, ****p < 0.0001; Two-way ANOVA followed by Tukey’s post hoc test. One single dot corresponds to one mouse, each in **a**–**c**. Average values are mean values ± SEM, each. **d** Pressure-volume (PV) loops of experiments as shown in **c**. Data are mean ± SEM calculated for each group. **e** Representative images of H&E-stained lung tissue sections from mouse lungs (BL6 WT and *Trpml3*^−*/*−^) exposed to Elastase or PBS showing the respective extent of airspace enlargements. Scale bar 100 µm. **f** Quantification of airspace enlargement as mean linear chord length. Lung tissue sections from 6–8 mice per group were analysed. Each dot corresponds to one biologically independent lung tissue sample. Average values are mean values ± SEM, each. *p < 0.05, ****p < 0.0001; One-way ANOVA followed by Tukey’s post hoc test. Source data are provided as a Source Data file.

We used two independently generated and differentially engineered *Trpml3*^−/−^ mouse models, *Mcoln3*^*tm1.2Hels*^ and *Mcoln3*^*tm1.1Jga*^^13,16^ to investigate the effects of the loss of TRPML3 on lung function. We first performed lung function measurements using the forced oscillation technique (FlexiVent/SCIREQ, snapshot perturbation). Both *Trpml3*^−/−^ mouse models showed a reduction of elastance of the whole respiratory system (elastance (E) captures the elastic rigidity or the stiffness of the lungs), whereas compliance was increased (compliance (C) captures the ease with which the lungs can be extended) (Fig. 2a, b). Such changes in elastance and compliance are major hallmarks of emphysema and are in accordance with observations made in the elastase-induced emphysema mouse model. To investigate the decline in lung function and the progression in airspace enlargement under the diseased condition we made use of this well-established elastase-induced emphysema mouse model^17,18^. The instilled porcine pancreatic elastase degrades elastin fibers in the lung tissue, leading to the destruction of alveolar walls, enlarges airways and reduces surface area^19^. These changes result in altered lung function parameters, such as increased compliance and decreased elastance^17^. When comparing PBS (control buffer) and elastase (20 U/kg) treated WT and *Trpml3*^−*/*−^ mice, we found a further enhanced emphysematous phenotype in *Trpml3*^−/−^ mice compared to WT mice. Thus, compliance and elastance showed the most prominent changes in the *Trpml3*^−*/*−^ elastase-treated group (Fig. 2c). In the more sophisticated constant-phase model (primewave-8 perturbation) (Fig. 2c), a further reduction of tissue elasticity (H; reflects the energy conservation in the alveoli) in elastase-treated *Trpml3*^−/−^ compared to elastase-treated WT mice was found. The value for inspiratory capacity (IC), which is the sum of TV (tidal volume) and IRV (inspiratory reserve volume = maximal volume that can be inhaled from the end-inspiratory level) was significantly increased in *Trpml3*^−*/*−^ mice in the elastase-treated group, but not in the PBS treated group (Fig. 2c). Likewise, the pressure-volume (PV) loop analysis revealed a significant increase of MVC (maximal vital capacity = total lung capacity (TLC), abbreviated with A) in *Trpml3*^−/−^ mice in the elastase-treated group. The quasistatic compliance (Cst; reflects the static elastic recoil pressure of the lungs at a given lung volume) was significantly increased again only in *Trpml3*^−/−^ mice in the elastase-treated group, but not in the PBS treated group (Fig. 2c). The pressure-volume loops showed shifts towards larger volumes in *Trpml3*^−/−^ mice compared to WT mice under both basal and elastase treatment, characteristic for emphysema and a direct result of the destruction of pulmonary architecture, making emphysematous airways more prone to collapse during expiration (Fig. 2d). The quantitative histological analysis of WT and *Trpml3*^−/−^ mouse lung samples under basal conditions and after elastase treatment revealed increased airspace enlargements, which were more pronounced in *Trpml3*^−/−^ mice compared to WT mice (Fig. 2e, f). In summary, both *Trpml3*^−*/*−^ mouse strains show impaired lung function parameters, which are in accordance with an emphysema phenotype. Exacerbation of this phenotype after elastase treatment was found to be more pronounced in *Trpml3*^−/−^ compared to WT mice.

### Selective activation of mTRPML3 with small-molecule agonist ML3-SA1

**Fig. 3:**
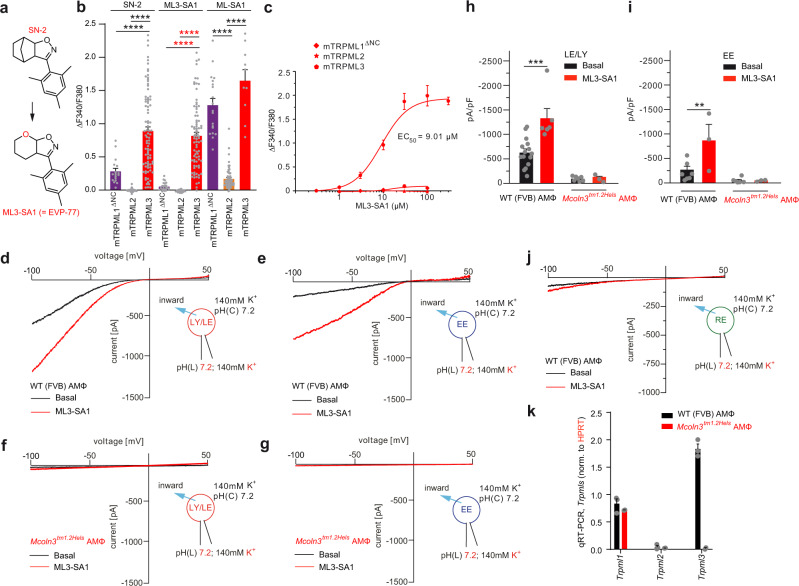
Effect of isoform-selective TRPML3 agonist ML3-SA1 on mouse TRPML1, 2, 3 in HEK293 cells and functional characterization of endogenous TRPML3 currents in murine AMΦ organelles. **a** Chemical structures of SN-2 and ML3-SA1 (= EVP-77). **b** Fura-2 calcium imaging experiments using HEK293 cells expressing human or murine TRPML1(NC), TRPML2, or TRPML3, respectively, indicating the specific levels of activation. Channels were stimulated with either SN-2, ML3-SA1, or ML-SA1 (10 µM, each). Shown are average values (mean ± SEM). Each dot represents one biologically independent experiment with 10–20 cells, each. ****p < 0.0001; Two-way ANOVA followed by Tukey’s post hoc test. **c** Dose-response curves obtained from experiments as described in **b** using ML3-SA1 on murine TRPML1-3 expressing HEK293 cells. **d**–**g** Representative currents from YM201636-enlarged LE/LY or Wort./Lat.B-enlarged EE isolated from murine (WT or *Trpml3*^−*/*−^) primary AMΦ, elicited by an application of 10 µM ML3-SA1, respectively. **h**–**i** Statistical summary of data shown in **d**–**g**. Each dot corresponds to one biologically independent experiment. Average values are mean values ± SEM, each. In all experiments, conditions were set to evoke maximal TRPML3 current activity (neutral pH, low sodium). **p < 0.01, ***p < 0.001; One-way ANOVA followed by Tukey’s post hoc test. **j** Representative currents from vacuolin-enlarged Tf+ RE isolated from murine (WT or *Trpml3*^−*/*−^) primary AMΦ, elicited by an application of 10 µM ML3-SA1, respectively. **k** qRT-PCR results for *Trpml1*, *Trpml2*, and *Trpml3* in AMΦ normalized to HPRT (*n* = 3 biologically independent experiments, each. Average values are mean values ± SEM, each). Source data are provided as a Source Data file.

The widely used compound ML-SA1 is a small molecule that can activate all three human TRPML channel isoforms and TRPML1 and 3 in mouse. Likewise, the endogenous TRPML channel activator phosphoinositol 3,5-bisphosphate (PI(3,5)P_2_) activates all three TRPML channels and, in addition also activates the TRPML-related endolysosomal cation channels TPC1 and TPC2^20–22^. To improve the selectivity profiles of currently available small molecule TRPML activators, we have recently generated >50 derivatives of the small molecule TRPML agonist SN-2^14,23^. The compounds were tested in fura-2 calcium imaging and in early endosome (EE) and late endosome (LE)/lysosome (LY) patch-clamp experiments^14^. One compound that has not been published previously, ML3-SA1 (=EVP-77) shows improved selectivity for mTRPML3 (mouse TRPML3) over mTRPML1 and mTRPML2 compared to SN-2 (Fig. 3a–c) and was therefore used for the following selective characterization of TRPML3 currents in mouse AMΦ.

### Endogenous TRPML3 is found in EE and LE/LY isolated from AMΦ

Using ML3-SA1, endogenous TRPML3 currents were detectable in both EE and LE/LY isolated from AMΦ, but not in recycling endosomes (RE) (Fig. 3d–j). In LE/LY isolated from WT AMΦ TRPML3 currents could be detected with ML3-SA1 showing a maximal effect in the presence of high luminal potassium at pH 7.2^24^. In LE/LY isolated from *Trpml3*^−*/*−^ AMΦ ML3-SA1 induced currents were completely absent, but TRPML1/TPC-like currents could still be elicited (positive control; Fig. S3a). Similarly, in EE we found maximal TRPML3 currents in the presence of high luminal potassium at pH 7.2^24^. Again, no currents were detectable in EE isolated from *Trpml3*^−*/*−^ AMΦ. In contrast, TPC-like currents were still detectable in EE isolated from *Trpml3*^−*/*−^ AMΦ (positive control; Fig. S3b). In line with these data qRT-PCR analysis confirmed absence of TRPML3 in *Trpml3*^−*/*−^ AMΦ (Fig. 3k). In contrast to EE and LE/LY no endogenous TRPML3 activity could be detected in the plasma membrane of AMΦ (Fig. S3c, d). We also assessed TRPML3 current densities in LE/LY isolated from GFP + (TRPML3 +) versus GFP- (TRPML3-) AMΦ from *Trpml3*^IRES-Cre/eR26-τGFP^ mice, revealing a clear correlation between GFP fluorescence and TRPML3 activity (Fig. S3e-i). Together, these data demonstrate expression of TRPML3 in EE and LE/LY but not in RE of AMΦ. Of note, TRPML3 is particularly active at more neutral pH (physiological pH in EE is 6-7), while being less active at pH 4–5 (LE/LY)^24^, pointing to an important role in EE under physiological conditions.

### Increased MMP-12 levels in *Trpml3*^−*/*−^ bronchoalveolar lavage fluid (BALF) and in AMΦ supernatant

**Fig. 4:**
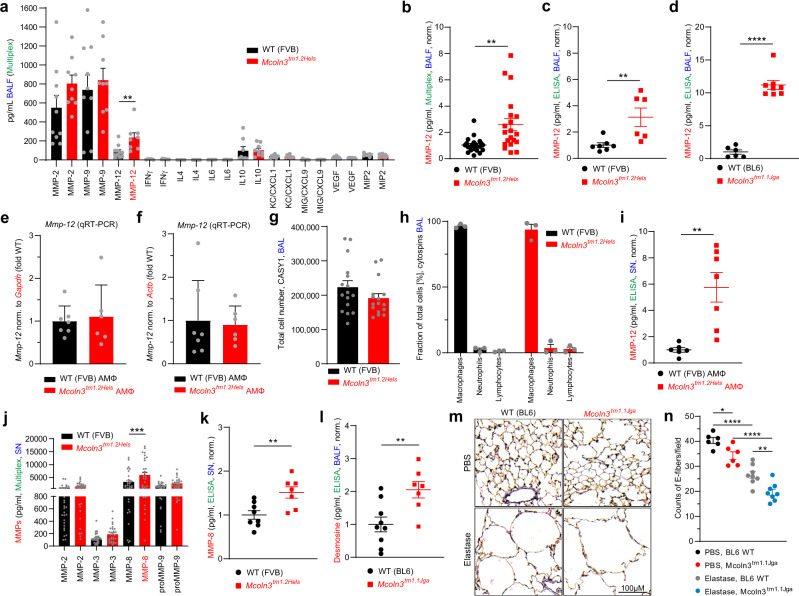
Increased MMP-12 levels in WT and *Trpml3*^−*/*−^. **a** Quantification of the levels of different chemokines/cytokines and MMPs in BALF isolated from 4-month old WT and *Trpml3*^−*/*−^ mice using Multiplex analysis. **b** Repeated Multiplex analysis of MMP-12 levels in BALF. **c**, **d** MMP-12 quantification in BALF isolated from 4-month old WT and *Trpml3*^−*/*−^ mice using ELISA. One single dot corresponds to BALF from one mouse, each in **c**, **d**. **e**, **f** qRT-PCR data showing mRNA expression levels of *Mmp-12* in AMΦ (WT and *Trpml3*^−*/*−^). **g** Quantification of total cell numbers in BALF using the CASY1 cell counter. **h** Quantification of cell numbers in BALF using morphological criteria on May-Grünwald-Giemsa-stained cytospins. **i** MMP-12 quantification in the supernatant of cultured AMΦ isolated from 4-month old WT and *Trpml3*^−*/*−^ mice using ELISA. One single dot corresponds to the AMΦ SN from one well, each. Statistical analysis of datasets a-i was performed by using Student’s *t* test, unpaired, two-tailed (**p < 0.01, ****p < 0.0001). **j**, **k** MMP quantification in the supernatant of cultured AMΦ isolated from 4-month old WT and *Trpml3*^−*/*−^ mice using Multiplex and ELISA. One single dot corresponds to the AMΦ SN from one well, each. Two-way ANOVA followed by Tukey’s post hoc test; ***p < 0.001 (**j**) or Student’s *t* test, unpaired, two-tailed; **p < 0.01 (**k**). **l** Desmosine ELISA of BALF isolated from WT and *Trpml3*^−*/*−^ mice. One single dot corresponds to BALF from one mouse. Student’s *t* test, unpaired, two-tailed; **p < 0.01. **m** Verhoeff-Van Gieson (VVG) staining of formalin-fixed, paraffin-embedded lung sections of female, 4-month old WT or *Trpml3*^−*/*−^ mice (*Mcoln3*^*tm.1.1Jga*^) treated either with PBS or porcine pancreatic elastase. Elastic fibers are stained blue-black, collagen appears red, and other tissue elements yellow. Scale bar 100 µm. **n** Quantification of elastin fibers as counts per field in VVG stained lung tissue sections from 6–8 mice per group. One dot corresponds to the mean count of elastin fibers in 8-10 fields of view per mouse lung. *p < 0.05, ****p < 0.0001; One-way ANOVA followed by Tukey’s post hoc test. In all figures, each single dot corresponds to one biologically independent sample. Data are mean ± SEM. Source data are provided as a Source Data file.

To better understand the mechanism underlying the observed changes in lung function in *Trpml3*^−*/*−^ mice and to examine whether there is a direct link between TRPML3 expression/function in AMΦ and the observed emphysema phenotype, we performed different assays using BALF samples and AMΦ isolated from *Trpml3*^−*/*−^ and WT mice. First, we analysed the levels of secreted inflammatory mediators in BALF and in the supernatant (SN) of AMΦ, and in a second step, we performed a range of cell biological experiments including lysosomal pH, endo- and exocytosis measurements. The analysis of inflammatory mediator levels in BALF revealed that MMP-12 levels are significantly increased in BALF isolated from *Trpml3*^−*/*−^ compared to WT mice (Fig. 4a–d), while other inflammatory mediators such as interleukins and cytokines were not altered significantly. Experiments were performed in both Multiplex and ELISA format with both *Trpml3*^−*/*−^ mouse models, *Mcoln3*^*tm1.2Hels*^ and *Mcoln3*^*tm1.1Jga*^. In contrast, transcription of MMP-12 was normal as demonstrated by qRT-PCR analysis (Fig. 4e, f). The average total cell numbers and the average numbers of macrophages, neutrophils, and lymphocytes were found to be comparable in WT and *Trpml3*^−*/*−^ samples (Fig. 4g, h). Importantly, MMP-12 levels in the supernatant (SN) of cultured *Trpml3*^−*/*−^ AMΦ were increased compared to WT (Fig. 4i), suggesting that the changes seen in BALF were indeed due to functional changes in AMΦ. We also assessed other MMPs in AMΦ SN. MMP-2 and MMP-9 had not been found to be significantly changed in BALF using Multiplex (Fig. 4a). This was confirmed by SN measurements. Again, MMP-2, MMP-9, and additionally also MMP-3 were not significantly increased (Fig. 4j). This is in agreement with MMP-2, 3, and 9 not being expressed by AMΦ (Fig. S4). By contrast, MMP-8 levels were significantly increased in *Trpml3*^−*/*−^ compared to WT AMΦ SN using Multiplex, which we subsequently confirmed by using ELISA (Fig. 4j, k). MMP-8 is also called neutrophil collagenase or collagenase 2. It promotes normal neutrophil apoptosis and clearance, resulting in dampened inflammation. Disease-relevant roles in cancer and inflammatory arthritis have been reported^25^. Evidence for a relevant role in emphysema/COPD development is less established for MMP-8 compared to MMP-12, in particular in the mouse model^26^. Besides, MMP-12 expression is strongly linked to the different macrophage populations in the lung, including AMΦ, while MMP-8 is most strongly linked to neutrophils (Fig. S4). In the following, we therefore focused on MMP-12. To further validate the role of MMP-12 we assessed the levels of desmosine (biomarker for elastin degradation) in BALF using ELISA and we performed Verhoeff stainings to demonstrate elastic tissue atrophy and loss of elastic fibers (Fig. 4l–n). In accordance with the increased MMP-12 levels, desmosine levels were increased and the Verhoeff stainings revealed a reduction in elastin. The unchanged MMP-12 transcription combined with the increase in BALF and cultured AMΦ SN, while cell numbers were comparable pointed to a potential defect in endocytosis, endolysosomal trafficking and/or exocytosis/secretion of MMP-12 in *Trpml3*^−*/*−^ AMΦ. We next focused on possible alterations in the early endosomal pathway where TRPML3 is highly active under physiological conditions as outlined above (low pH blocks TRPML3 activity while more neutral pH increases activity)^24^.

### Defects in early endosomal trafficking and endocytosis in *Trpml3*^−*/*−^ AMΦ

**Fig. 5:**
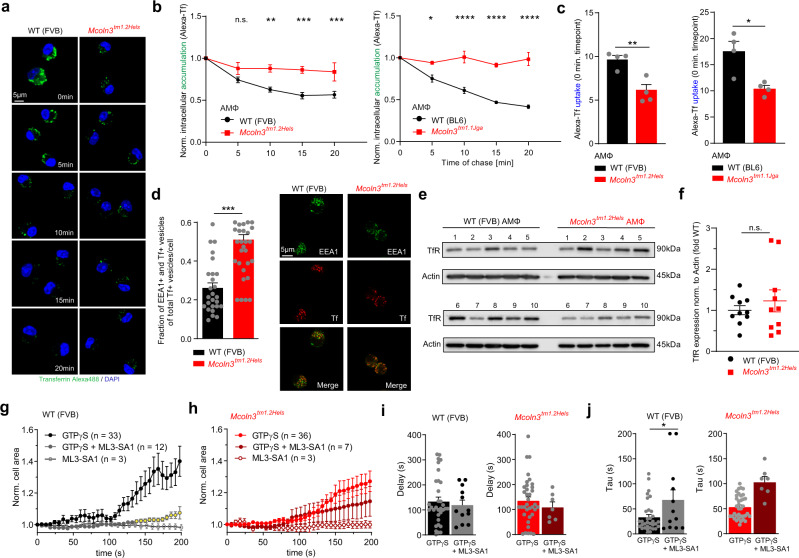
Early endosomal trafficking in WT and *Trpml3*^−*/*−^ AMΦ. **a**, **b** Transferrin (Tf) trafficking assay showing the decrease of Tf fluorescence in AMΦ (WT and *Trpml3*^−*/*−^) within 20 min after the pulse with Tf-AlexaFluor488 (Tf accumulation). Mean ± SEM, 4 biologically independent experiments, each. *p < 0.05, **p < 0.01, ***p < 0.001, ****p < 0.0001; Two-way ANOVA followed by Bonferroni’s post hoc test. **c** Tf fluorescence in AMΦ (WT and *Trpml3*^−*/*−^) after 20 min pulse with Tf-AlexaFluor488 (0 min timepoint, measures Tf uptake). Mean ± SEM, four biologically independent experiments. *p < 0.05, **p < 0.01, Student’s *t* test, unpaired, two-tailed. **d** Representative confocal images and quantification of the colocalization of EEA1 and Tf in AMΦ (WT and *Trpml3*^−*/*−^). Statistical analysis was performed using Student’s *t* test, unpaired, two-tailed. Mean ± SEM, three biologically independent experiments. *p < 0.05, ***p < 0.001. **e**, **f** TfR expression analysis using Western blot. **e** Shown are two independent WB blots for TfR (90 kDa) and ß-Actin (45 kDa; loading control) using 5 WT and 5 *Trpml3*^−*/*−^ AMΦ lysates on each blot. **f** Quantification of WB data as shown in **e**. TfR protein was normalized to ß-Actin and values from *Trpml3*^−*/*−^ AMΦ were normalized to WT AMΦ. One single dot corresponds to one mouse, each (mean ± SEM). **g**, **h** Whole-cell patch-clamp experiments to determine membrane capacitance (measure of cell surface area). GTPγS induces an increase in surface area. Co-application of ML3-SA1 significantly reduces the effect of GTPγS in WT AMΦ, but not in *Trpml3*^−*/*−^ AMΦ ( = loss of membrane surface). Significance: GTPγS vs. GTPγS + ML3-SA1 from 140 to 150 sec *, from 152 to 156 sec **, from 158 to 172 sec ***, from 174 to 194 **, then till 200 sec *** (yellow dots). *p < 0.05, **p < 0.01, ***p < 0.001; two-way ANOVA followed by Tukey’s multiple comparisons test. Shown are mean values ± SEM, *n* (in parentheses) = biologically independent experiments. **i**, **j** Bar diagrams (mean ± SEM) showing the parameters *Tau* (= time until 2/3 of the maximum amplitude is reached) and *Delay* (= time until capacitance changes). *p < 0.05, Student’s *t* test, unpaired, two-tailed. Source data are provided as a Source Data file.

**Fig. 6:**
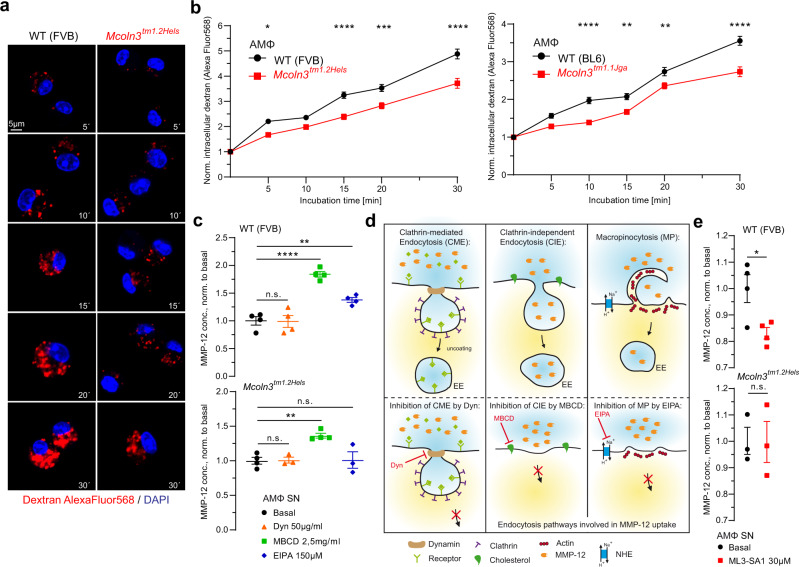
Endocytosis in WT and *Trpml3*^−*/*−^ AMΦ. **a** Shown are representative confocal images obtained from endocytosis experiments using dextran coupled to Alexa Fluor 568. Images show AMΦ (WT vs. *Trpml3*^−*/*−^) that have been pulsed with fluorescently labelled dextran for different time periods. Scale bar 5 µm. **b** Quantification of dextran uptake showing significantly decreased rates of endocytosis in *Trpml3*^−*/*−^ AMΦ compared to WT AMΦ at various time points. A sum of at least 130 cells were analysed per timepoint and genotype deriving from five biologically independent experiments for both *Trpml3*^−*/*−^ lines (*Mcoln3*^*tm1.2Hels*^ and *Mcoln3*^*tm1.1Jga*^), respectively. Data are mean ± SEM. *p < 0.05, **p < 0.01, ***p < 0.001, ****p < 0.0001; Two-way ANOVA followed by Bonferroni’s post hoc test. **c** Effect of different endocytosis inhibitors on MMP-12 levels in WT and *Trpml3*^−*/*−^ AMΦ supernatants (SN). *p < 0.05, **p < 0.01, ****p < 0.0001; One-way ANOVA followed by Dunnett’s post hoc test. One single dot corresponds to the AMΦ SN from one well, each. 11 WT and 11 *Trpml3*^−*/*−^ mice were lavaged to obtain the number of cells for all wells. Data are mean ± SEM. **d** Cartoon showing endocytosis of MMP-12 via three different endocytosis pathways (CME, CIE, MP) and the effect of endocytosis inhibitors on MMP-12 uptake: According to the results shown in (**c**) the MMP-12 uptake in AMΦ corresponds to CIE and MP, resulting in higher concentrations of MMP-12 in the extracellular fluid after inhibition of these pathways. CME seems to be not involved. **e** Effect of the selective TRPML3 agonist ML3-SA1 (incubation o.n., 30 µM) on MMP-12 levels in WT and *Trpml3*^−*/*−^ AMΦ supernatants (SN). *p < 0.05, Student’s *t* test, unpaired, two-tailed. One single dot corresponds to the AMΦ SN from one well, each. 5 WT and 5 *Trpml3*^−*/*−^ mice were lavaged to obtain the appropriate number of cells for all wells. Data are mean ± SEM. Source data are provided as a Source Data file.

To assess early endosomal trafficking we probed AMΦ with fluorescent transferrin (Tf). We found that both uptake and trafficking of transferrin through the early endosomal system were reduced or delayed in *Trpml3*^−*/*−^ AMΦ (Fig. 5a–c) and that colocalization with the early endosomal marker EEA1 was increased, suggesting a retention of transferrin in EE (Fig. 5d). At the same time expression of transferrin receptor (TfR) was unchanged in *Trpml3*^−*/*−^ compared to WT AMΦ (Fig. 5e, f). We next performed electrophysiological measurements of membrane capacitance via the whole-cell patch-clamp technique as an estimate of cell surface area as previously reported^27,28^, to uncover potential differences caused by changes in global exo- and/or endocytosis rates (Fig. 5g–j). In WT AMΦ we found changes in normalized cell surface area after stimulation with GTPγS (guanosine 5′-O-[gamma-thio]triphosphate). These changes were not significantly different from changes measured in *Trpml3*^−*/*−^ AMΦ. However, when co-applying the TRPML3-selective agonist ML3-SA1 effects of GTPγS were reduced in WT AMΦ, whereas in *Trpml3*^−*/*−^ AMΦ GTPγS effects were not significantly altered. These data suggested that TRPML3 activation either reduces exocytosis or increases endocytosis. To evaluate endocytosis further we used fluorescent dextran (10 kDa) (Fig. 6a, b) and found reduced bulk endocytosis rates in *Trpml3*^−*/*−^ AMΦ isolated from both *Trpml3*^−*/*−^ mouse models. We next used blockers of endocytosis, specifically Dynasore (Dyn) for clathrin-mediated endocytosis (CME), methyl-β-cyclodextrin (MBCD) for caveolae-mediated endocytosis (clathrin-independent endocytosis (CIE)), and 5-(N-ethyl-N-isopropyl) amiloride (EIPA) for macropinocytosis (CIE) to assess their effects on MMP-12 levels in AMΦ SN (Fig. 6c, d). We found that only blockers of CIE/macropinocytosis but not CME increased the MMP-12 levels in the SN of AMΦ, suggesting that MMP-12 can be endocytosed via CIE/macropinocytosis by AMΦ and that CIE/macropinocytosis blockage increases extracellular MMP-12 levels. Dyn had no effect on MMP-12 levels in WT cells and expectedly was also found to have no effect on MMP-12 in *Trpml3*^−*/*−^ AMΦ. MBCD still increased MMP-12 levels in *Trpml3*^−*/*−^ AMΦ compared to basal, suggesting TRPML3 independent effects of MBCD on MMP-12 endocytosis. By contrast, the macropinocytosis blocker EIPA did not increase MMP-12 levels in *Trpml3*^−*/*−^ AMΦ anymore compared to basal, suggesting a TRPML3-dependent effect and a role of TRPML3 in macropinocytosis of MMP-12 in AMΦ (the EIPA effect in WT versus *Trpml3*^−*/*−^ AMΦ was statistically also not significant). Finally, we tested the selective mTRPML3 agonist ML3-SA1 (a selective TRPML3 blocker is currently not available) and found decreased MMP-12 levels in the SN of AMΦ while no compound effect was observed in *Trpml3*^−*/*−^ AMΦ SN compared to control (Fig. 6e). To exclude any cytotoxic effects of long-term exposure to the agonist, we performed LDH (lactate dehydrogenase) cytotoxicity assays with 30 µM ML3-SA1 overnight (Fig. S5).

### Lysosomal exocytosis, TRPML1 activity, lysosomal pH, and autophagy in *Trpml3*^−*/*−^ AMΦ

**Fig. 7:**
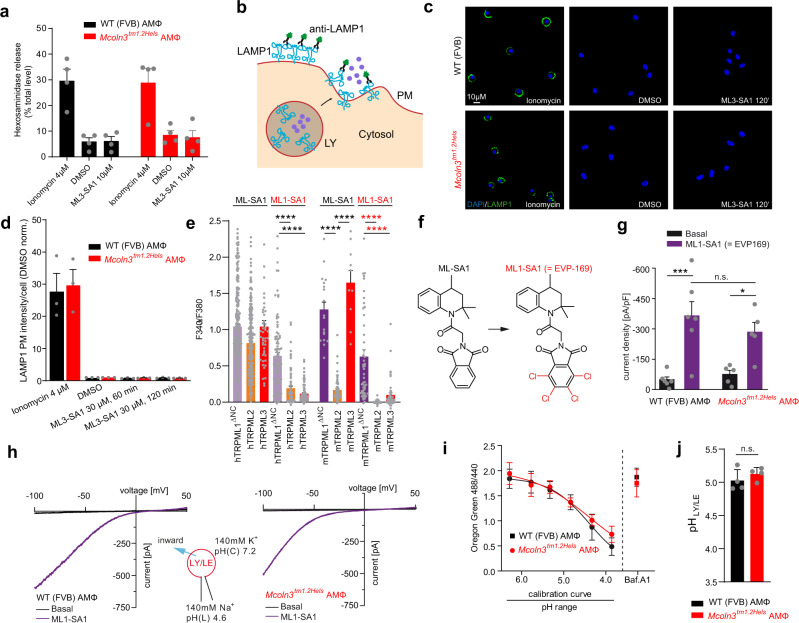
Lysosomal exocytosis, pH and TRPML1 activity in WT and *Trpml3*^−*/*−^ AMΦ. **a** Lysosomal exocytosis experiments measuring hexosaminidase release from WT and *Trpml3*^−*/*−^ AMΦ. Maximum effects were obtained with ionomycin (4 µM). TRPML3 activator ML3-SA1 elicited no significant effects in both WT and *Trpml3*^−*/*−^ AMΦ. Each dot corresponds to one biologically independent experiment. Average values are mean values ± SEM, each. **b** Cartoon illustrating LAMP1 translocation assay shown in **c**, **d**. Upon lysosomal exocytosis the lysosomal protein LAMP1 is detected on the plasma membrane (PM) by anti-LAMP1 followed by Alexa Fluor 488-conjugated secondary antibody. **c** Representative images of LAMP1 translocation assay using WT and *Trpml3*^−*/*−^ AMΦ. Shown are results obtained after 120 min treatment with DMSO, ML3-SA1 (30 µM), or 10 min treatment with ionomycin (4 µM). Scale bar 10 µm. **d** Quantification of experiments as shown in **c** (mean ± SEM from 3 biologically independent experiments, each). **e** Fura-2 calcium imaging experiments using HEK293 cells expressing human or murine TRPML1(NC), TRPML2 or TRPML3, respectively, indicating the specific levels of activation. Channels were stimulated with either ML1-SA1 ( = EVP-169) or ML-SA1 (10 µM, each). Shown are average values (mean ± SEM). Each dot represents one biologically independent experiment with 10–20 cells, each. ****p < 0.0001; Two-way ANOVA followed by Tukey’s multiple comparisons test. **f** Chemical structures of ML-SA1 and its derivative ML1-SA1 ( = EVP-169). **g**, **h** Quantification (**g**) and representative currents (**h**) from YM201636-enlarged LE/LY isolated from WT or *Trpml3*^−*/*−^ AMΦ, elicited by an application of 10 µM ML1-SA1. Each dot corresponds to one biologically independent experiment. Average values are mean values ± SEM, each. *p < 0.05, ***p < 0.001; One-way ANOVA followed by Tukey’s post hoc test. **i** Results obtained from endolysosomal pH measurements using WT or *Trpml3*^−*/*−^ AMΦ. Measurements were performed by ratiometric fluorescence imaging with Oregon Green^22,71^. Data are mean ± SD. **j** Mean endolysosomal pH values (mean ± SD) in WT and *Trpml3*^−*/*−^ AMΦ were calculated using the calibration curves presented in **i** (*n* = 4, each). Source data are provided as a Source Data file.

The membrane capacitance measurements suggested that TRPML3 activation may alternatively affect exocytosis. To investigate lysosomal exocytosis, we performed beta-hexosaminidase release and lysosomal-associated membrane protein type 1 (LAMP1) plasma membrane translocation assays. In beta-hexosaminidase release assays, we found no differences between WT and *Trpml3*^−*/*−^ AMΦ when stimulated with the selective mTRPML3 agonist ML3-SA1 or when treated with DMSO, while the positive control ionomycin increased hexosaminidase release (Fig. 7a). In LAMP1 translocation assays, we likewise found no differences between WT and *Trpml3*^−*/*−^ AMΦ when stimulated with the selective mTRPML3 agonist ML3-SA1 compared to DMSO control. The positive control ionomycin increased LAMP1 translocation to the plasma membrane (PM) (Fig. 7b–d). These data suggested lysosomal exocytosis neither to be increased in the absence of TRPML3 nor to be affected by selective TRPML3 activation. Next, we assessed potential changes in the activity of TRPML1 due to the loss of TRPML3. Notably, TRPML1 activation increases lysosomal exocytosis^10^. To exclusively measure TRPML1 activity, we made use of a hitherto unpublished agonist, ML1-SA1 (=EVP169), showing selectivity for TRPML1 over TRPML2 and TRPML3 (Fig. 7e, f). We assessed TRPML1 activity in WT and *Trpml3*^−*/*−^ AMΦ using the endolysosomal patch-clamp technique but found no differences upon ML1-SA1 application, suggesting normal TRPML1 channel activity in *Trpml3*^−*/*−^ AMΦ (Fig. 7g, h). This was further corroborated by qRT-PCR experiments, revealing expression of TRPML1 not to be altered in WT versus *Trpml3*^−*/*−^ whole lung or AMΦ samples (Fig. S2b and Fig. 3k). Taken together, a role of TRPML3 in the release of MMP-12 via lysosomal exocytosis from LY appears unlikely and the increased MMP-12 levels in BALF or *Trpml3*^−*/*−^ AMΦ supernatant cannot be explained with a defect in the release from LY as lysosomal exocytosis is not increased in *Trpml3*^−*/*−^. Since TRPML3 was also found to be largely absent from RE as confirmed by patch-clamp electrophysiology (Fig. 3j), a role in the release from RE seems unlikely as well. Next, lysosomal pH was measured to assess any defects in general lysosomal function based on luminal pH changes. However, lysosomal pH in *Trpml3*^−*/*−^ was likewise not different from WT AMΦ (Fig. 7i, j). Further, we tested the effects of loss of TRPML3 on autophagy. Autophagy can reportedly play a dual role in COPD. Thus, increased autophagy is associated with exacerbated COPD pathogenesis by promoting epithelial cell death, while defective autophagy in AMΦ was postulated to promote recurrent infections in COPD patients^29–33^. We tested LC3-I versus LC3-II expression in WT and *Trpml3*^−*/*−^ AMΦ using Western blot analysis with or without bafilomycin treatment (Fig. S6a, b). While we found a reduction of LC3-II in both mouse models (*Mcoln3*^*tm1.2Hels*^ and *Mcoln3*^*tm1.1Jga*^), the bafilomycin experiment indicates that *Trpml3*^−*/*−^ AMΦ are still autophagy competent. However, we cannot exclude defects in the biogenesis of autophagosomes. Thus, loss of TRPML3 could play a further exacerbating role, increasing the severity and progression of emphysema/COPD, under infectious conditions. Next, we tested surfactant protein (SP) and (phospho)lipid levels in BALF. The most abundant surfactant protein (SP) is SP-A, expressed by alveolar type II cells, club cells and submucosal gland cells. SP-A has been correlated with lung fibrosis and genetic defects in surfactant protein A2 are associated with pulmonary fibrosis and lung cancer^34^. SP-B is also expressed by alveolar type II cells and club cells. SP-C is exclusively expressed by alveolar type II cells. Interestingly, SP-D deficient mice have an emphysema phenotype and macrophages from SP-D deficient mice produce more MMP-2, -9, and -12^35^. We therefore tested levels of SP-D using ELISA in BALF and found that SP-D levels were not significantly different in WT versus *Trpml3*^−*/*−^ BALF (Fig. S7a). The largest proportion of pulmonary surfactant accounts for lipids, in particular, ~80% phosphatidylcholine (PC), ~10% phosphatidylglycerol (PG), ~10% cholesterol (CE) and small amounts of phosphatidylinositol (PI), phosphatidylserine (PS), phosphatidyl-ethanolamine (PE), triglycerides (TG), and free fatty acids (FFA)^36,37^ (Fig. S7b). Decreased surfactant lipids correlate with lung function and COPD. Thus, the surfactant lipidome can be substantially altered in subjects with COPD, and decreased availability of phospholipids correlates with decreased pulmonary function^38,39^. Therefore, we analysed the phospholipid content of BALF and found no changes in WT compared to *Trpml3*^−*/*−^ BALF samples (Fig. S7c), same as for several, major PC variants (Fig. S7d). Finally, we examined NF-κB expression levels. The transcription factor NF-κB (nuclear factor kappaB) plays an important role in airway pathology, including COPD by regulating the expression of chemokines and cytokines, and higher levels of NF-κB have been observed in bronchial biopsies and inflammatory cells of COPD patients^40^. We probed the NF-κB pathway in WT and *Trpml3*^−*/*−^ AMΦ by using one of the most potent inducers of the NF-κB signaling pathway, TNFα (tumor necrosis factor alpha). No alteration in the NF-κB signaling pathway was detectable in absence of TRPML3, while TNFα was able to produce NF-κB response in both WT and *Trpml3*^−*/*−^ AMΦ, as shown by the phosphorylation on serine s932 of NF-κB p105 and s536 of NFKB p65 (Fig. S7e, f).

**Fig. 8:**
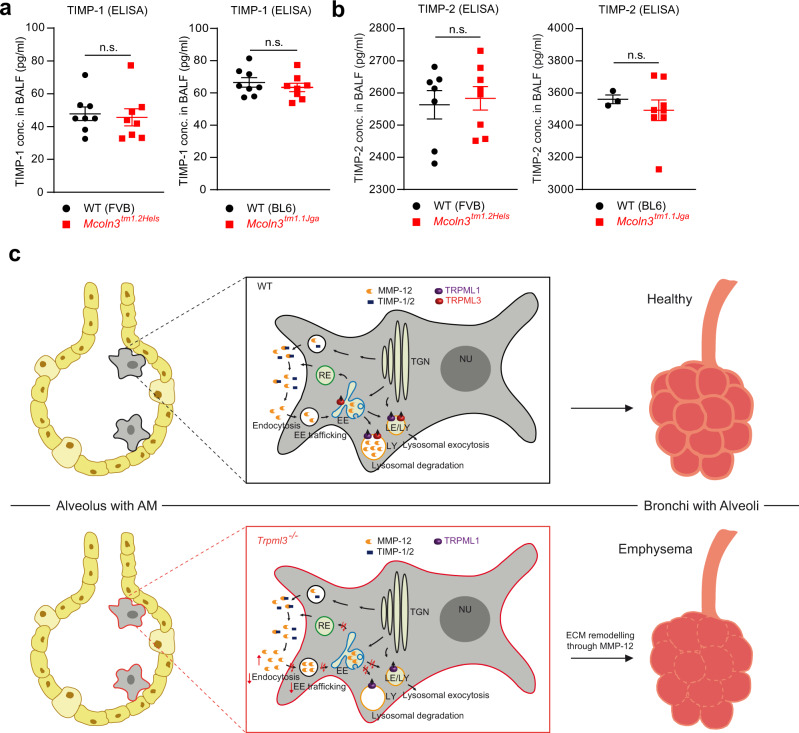
TIMPs in WT and *Trpml3*^*−/−*^ BALF and schematic of emphysema development in *Trpml3*^*−/−*^ lungs. **a**, **b** TIMP-1 and TIMP-2 levels in BALF obtained from WT and *Trpml3*^*−/−*^ mice measured by ELISA. Data are mean ± SEM collected from up to 8 mice per genotype per mouse line. Statistical analysis was performed using Student’s *t* test, unpaired, two-tailed. One single dot corresponds to one mouse, each in **a**, **b**. **c** Scheme showing the mechanism of emphysema development in *Trpml3*^*−/−*^ mouse lungs. In WT lungs the amount of MMP-12 outside the AMΦ is regulated by TIMP-1/2, as well as endocytosis of MMP-12 and lysosomal degradation. We observed increased MMP-12 levels in BALF and lower endocytosis rates in *Trpml3*^*−/−*^ AMΦ. Vice versa selective activation of TRPML3 resulted in reduced MMP-12 levels in BALF. Therefore, it is postulated that loss of TRPML3 results in extracellular matrix (ECM) remodeling and emphysema characterized by destruction of the alveolar walls as depicted. Source data are provided as a Source Data file.

In sum, these data suggest that the increase in MMP-12 in *Trpml3*^*-/-*^ BALF is the major driving force of the emphysema phenotype, caused by a combination of endocytosis and trafficking defects in the early endosomal pathway of AMΦ, leading to a backlog and congestion in the system and likely a reduced delivery of endocytosed MMP-12 to lysosomes for degradation. As consequence of the backlog MMP-12 accumulates in the extracellular matrix (ECM), with the potential to promote emphysema development. In the ECM inhibitors of metalloproteinases (TIMPs) are highly critical in controlling MMP activity^41,42^, including MMP-12^42–44^. The overabundance of MMPs versus TIMPs can lead to emphysema, while enhanced inhibition can contribute to fibrotic pulmonary disease. An imbalance between MMPs and TIMPs in favor of MMPs can lead to inappropriate extracellular matrix (ECM) loss, or conversely, an imbalance favoring TIMPs can abrogate MMP activity, leading to excess ECM deposition. We therefore assessed BALF levels of relevant TIMPs^45,46^. TIMP-1 was found to be unchanged in both *Trpml3*^*−/−*^ mouse models, compared to WT controls (Fig. 8a). Likewise, TIMP-2 BALF levels were unchanged (Fig. 8b), suggesting an overall imbalance of MMP-12 and TIMPs due to a dysfunction of endocytosis/reuptake of MMP-12 by *Trpml3*^*−/−*^ AMΦ (Fig. 8c).

### Tobacco smoke exposure further exacerbates the emphysema phenotype in *Trpml3*^*−/−*^ mice

**Fig. 9:**
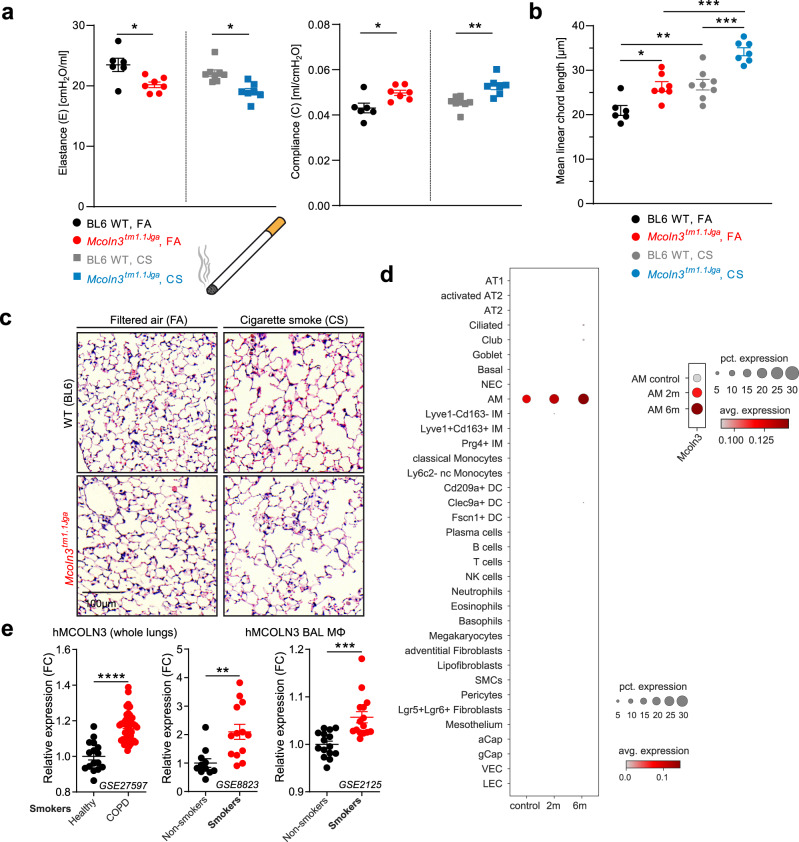
Effects of tobacco smoke exposure in WT and *Trpml3*^−*/*−^ mice (*Mcoln3*^*tm1.1Jga*^). **a** Lung function measurements were performed using the SCIREQs FlexiVent System in analogy to experiments shown in Fig. 2. Elastance and Compliance in *Trpml3*^−/−^ mice are changed in the direction of an emphysematous lung, both under filtered air (FA) and under cigarette smoke (CS). **b** Quantification of airspace enlargement as mean linear chord length. Lung tissue sections from 6–8 mice per group were analysed. Each dot corresponds to one biologically independent lung tissue sample. Average values are mean values ± SEM, each. *p < 0.05, **p < 0.01, ***p < 0.001; One-way ANOVA followed by Tukey’s post hoc test. **c** Representative images of H&E-stained lung tissue sections (as quantified in **b**) from mouse lungs (BL6 WT and *Trpml3*^*−/−*^) exposed to CS or FA showing the respective extent of airspace enlargements. Scale bar 100 µm. **d** Transcriptomics data of single-cell suspensions from female and WT whole mouse lungs that were exposed to FA or CS for 2 or 6 months. Dot plot shows the percentage of cells expressing *Mcoln3* using dot size and the average expression level of *Mcoln3* coded by color grading. **e** mRNA expression level of MCOLN3 in publicly available transcriptomics datasets obtained from the lungs of COPD patients with smoking history compared to healthy smokers (GSE27597), and in macrophages (MΦ) isolated from the bronchoalveolar lavage (BAL) of smokers compared to nonsmokers (GSE8823 and GSE2125), one single dot per person. Expression levels were normalized to the representative control groups. FC fold change. **p < 0.01, ***p < 0.001, ****p < 0.0001; two-tailed Mann-Whitney test. Source data are provided as a Source Data file.

Tobacco smoke exposure is one of the most intensively studied and most relevant causes of COPD. To corroborate the link between COPD and TRPML3 we performed lung function measurements, using again the forced oscillation technique introduced before. We treated *Trpml3*^−*/*−^ and WT mice for 2 months with either filtered air (FA; control) or cigarette smoke (CS) twice per day for 50 min (total particulate matter: 500 mg/m^3^) with 3 h breaks in between. Measurements of elastance (E) and compliance (C) revealed again, as observed in the elastase experiments before, the strongest phenotype exacerbation in *Trpml3*^−*/*−^ mice (Fig. 9a, b). In line with this, the quantitative histological analysis of WT and *Trpml3*^−*/*−^ mouse lung samples after cigarette smoke versus filtered air treatment revealed increased airspace enlargements, which were most pronounced in *Trpml3*^*−/−*^ mice treated with CS (Fig. 9c). Finally, transcriptomics analyses of single-cell suspensions from whole WT mouse lungs after FA versus CS exposure for different time intervals (2 and 6 months) revealed a higher number of AMΦ in the CS groups expressing TRPML3 compared to control (coded by dot size), as well as an upregulated average expression of TRPML3 (coded by colour grading) in AMΦ (Fig. 9d). In accordance with this, we found the relative expression of TRPML3 to be higher in samples from human smokers with COPD versus healthy smokers. Likewise, TRPML3 relative expression was higher in smokers compared to nonsmokers in two independent datasets (Fig. 9e), suggesting that in both smoke-exposed mice and humans, TRPML3 may be upregulated to counteract unbalanced levels of inflammatory mediators such as MMP12 via increased endocytosis. However, further analyses in COPD patients and smokers are needed to confirm these observations.

## Discussion

We show here a link between an endolysosomal cation channel, TRPML3, and the development of lung dysfunction and an emphysema-like phenotype in *Trpml3*^*−/−*^ mice due to the inability of *Trpml3*^*−/−*^ AMΦ to appropriately regulate MMP-12 levels in BALF. Albeit we cannot fully exclude that other MMPs e.g., MMP-8, or additional factors may also play a role, MMP-12 is mainly produced by AMΦ and has been convincingly demonstrated before to be involved in acute and chronic pulmonary inflammatory diseases associated with an intense airway remodeling, such as emphysema formation and COPD. When subjected to cigarette smoke WT but not MMP-12^−/−^ mice develop emphysema^9^. Furthermore, Haq et al.^47^ found a strong association of human MMP-12 single-nucleotide polymorphisms with severe to very severe COPD. Hunninghake et al.^48^ tested for an association between single-nucleotide polymorphisms in the *MMP-12* gene encoding MMP-12 and lung function in more than 8000 subjects. In sum, their data suggested that the minor allele of a SNP in *MMP-12* (rs2276109) is associated with a positive effect on lung function in children with asthma and in adults who smoke. This allele is also associated with a reduced risk of COPD in adult smokers. Broad-spectrum MMP inhibitors and more specific inhibitors for MMP-9/MMP-12 such as AZ11557272 or MMP-12 only, such as AS111793 or MMP408 provide significant protection against emphysema^49–53^, and both inflammatory processes and airspace enlargement in lung tissue can be reduced with MMP-12 inhibitors. While the role of MMP-12 in emphysema and COPD pathology is well established, it remains largely unclear what the molecular components are which regulate the secretion of MMP-12 and how clearance of excessive MMP-12 levels in the ECM/BALF is regulated. Intracellular MMP-12 processing is likewise not understood^54^. We found here that BALF levels of MMP-12 are strongly increased in two *Trpml3*^*−/−*^ mouse models compared to control mice. We also found an impairment in endolysosomal trafficking and endocytosis in *Trpml3*^*−/−*^ AMΦ. When endocytosis in cultured *Trpml3*^*−/−*^ and WT AMΦ was inhibited by CIE blockers, MMP-12 levels in AMΦ SN were increased, and EIPA, a blocker of macropinocytosis showed a TRPML3-dependent effect on MMP-12 endocytosis. Finally, the isoform-selective mTRPML3 agonist ML3-SA1 resulted in reduced MMP-12 levels in WT AMΦ SN but not *Trpml3*^*−/−*^ AMΦ SN, further corroborating a direct involvement of TRPML3.

So far, no other disease phenotypes have been demonstrated for *Trpml3*^*−/−*^ mice. Only TRPML double knockout mice (*Trpml1/Trpml3*^*−/−*^) were reported with an enterocyte/intestinal phenotype. Single knockouts were explicitly not affected, had normal intestinal anatomy and function, and normal growth rates^13^. To assess other possible organ defects with potential impact on the lung phenotype, we checked multiple parameters in serum, including markers for liver and kidney function (ALAT, ASAT, GLDH, cholesterol, urea, creatinine), glucose, triglycerides, protein, and LDH (lactate dehydrogenase) in 3- and 6-month-old mice. Furthermore, using ICP-MS we assessed potential abnormalities in Mg^2+^ levels in *Trpml3*^*−/−*^ mice (several organs, urine, feces, and serum were tested)^55^. Mg^2+^ deficiency can lead to emphysema as recently reported for TRPM6 knockout mice^55^. (Fig. S8–S11). Additional elements/trace metals were also tested using the same method without revealing significant differences (Fig. S10, S11). Length, body weight and organ to body weight ratios of several organs were monitored and found to be normal and in hematoxylin/eosin (HE) stainings no obvious differences were detectable (Fig. S12). White blood cell counts were also normal and not different between WT and *Trpml3*^*−/−*^ mice (tested at 3- and 6 months of age) (Fig. S9). A detailed FACS analysis of immune cell populations (monocytes, neutrophils, macrophages, and dendritic cells) in the bone marrow and spleen samples from 5-month-old WT and *Trpml3*^*−/−*^ mice after seven days in culture or directly after harvesting revealed no differences (Fig. S13). Besides, mice were housed in individually ventilated cages and not exposed to any pathogens associated with the respiratory system, ruling out that recurrent infections may have impacted lung function.

In sum, we show here that *Trpml3*^*−/−*^ mice are highly vulnerable to emphysema and COPD development. We further deliver a molecular rationale for the observed lung phenotype in *Trpml3*^*−/−*^ mice, we introduce TRPML3 as a regulator of MMP-12 levels in BALF, we provide a possible mechanism for cell entry of MMP-12, and we propose TRPML3 as a potential drug target for COPD and emphysema treatment.

## Method

All research performed complies with all relevant ethical regulations. Animals were used under animal protocols approved by the government (Regierung von Oberbayern, ROB-55.2-2532.Vet_02-17-170 and ROB-55.2-2532.Vet_02-18-6), and University of Munich (LMU) and the German Center for Lung Research (DZL) Institutional Animal Care Guidelines. Mice were housed in rooms maintained at constant temperature (20–24 °C) and humidity (45–65%) with a 12 hour light cycle. Animals were allowed food and water ad libitum.

### Lung function tests

Pulmonary function in mice was measured using a FlexiVent system running Flexiware software v7.6.4 (SCIREQ, Montréal, Canada). Mice were anesthetized with ketamine-xylazine, tracheostomized and connected to the FlexiVent system. Mice were ventilated with a tidal volume of 10 ml/kg at a frequency of 150 breaths/min in order to reach a mean lung volume similar to that of spontaneous breathing. Testing of lung mechanical properties, including dynamic compliance, elastance, tissue elasticity, inspiratory capacity, total lung capacity and quasistatic compliance was carried out by a software-generated script that took four readings per animal. For all experiments, female animals (4–5 months old) were used.

### Emphysema mouse model

Female WT vs. *Trpml3*^*−/−*^ mice (4–5 months old) were treated oropharyngeally with 20 U/kg body weight porcine pancreatic elastase (45124, Sigma). Control mice received a comparable volume of PBS. Development of emphysema was assessed by lung function measurements using the FlexiVent system 21 days after the application. Lung tissue was taken for histological analysis.

### Tobacco smoke experiments

Female WT vs. *Trpml3*^−/−^ mice (4–5 months old) were whole-body exposed to cigarette smoke (CS) of 500 mg/m^3^ total particulate matter (TPM) for 50 min twice per day for 2 months. CS was generated from 3R4F Research Cigarettes (Tobacco Research Institute, University of Kentucky) with filters removed and drawn into an exposure chamber via a membrane pump. TPM levels were monitored via gravimetric analysis of quartz fiber filters prior and after sampling air from the exposure chamber and measuring the total air volume^44^. CO concentrations in the exposure chamber were constantly monitored by using a GCO 100 CO Meter (Greisinger Electronic, Regenstauf, Germany) and reached values of 288 ± 74 ppm^44^. Control mice were kept in a filtered air (FA) environment. By the end of the 2-month FA/CS treatment, all animals were subjected to lung function analysis using the FlexiVent system. Lung tissue was taken for histological analysis.

### Lung tissue processing

Female mouse lungs were fixed at a constant pressure (20 cm fluid column) by intratracheal instillation of PBS buffered 6% paraformaldehyde (PFA). Left lung lobes were embedded into paraffin for histological analysis of hematoxylin and eosin (H&E) stained sections^56^ or for histological analysis of Verhoeff-van Gieson (VVG) stained sections using a staining kit from Morphisto (Cat. No. 18553). For quantification of elastin in the VVG stained lung tissue 8-10 fields of view per mouse lung were chosen randomly and the number of elastin fibers were counted in every field. The analysis was performed using an Olympus BX51 light microscope with a 40x lens.

### Quantitative morphometry

Design-based stereology was used to analyse sections using an Olympus BX51 light microscope equipped with a computer-assisted stereological toolbox (newCAST, Visiopharm) running Visopharm Integrator System (VIS) v.6.0.0.1765 software, on H&E-stained lung tissue sections as previously described^56^. Air space enlargement was assessed by quantifying mean linear chord length (MLI) on 30 fields of view per lung using the 20X objective. Briefly, a line grid was superimposed on lung section images. Intercepts of lines with alveolar septa and points hitting air space were counted to calculate MLI applying the formula MLI = ΣP_air_ × L(p)/ΣI_septa_ × 0.5. P_air_ are the points of the grid hitting air spaces, L(p) is the line length per point, I_septa_ is the sum of intercepts of alveolar septa with grid lines^44^.

### Single-cell transcriptomics

Single-cell suspensions from whole mouse lung (C57BL/6) were prepared and used for single-cell RNA sequencing using the Dropseq technique followed by single-cell data analysis^15,56^. No new scRNA-seq data on WT mouse lungs were generated in this manuscript. The scRNA-seq data set in Fig. 1h and Fig. S1 encompasses 14,813 cells from mouse whole lungs published in Angelidis et al. (2019)^15^. We retrieved the data from Gene Expression Omnibus under the accession number GSE124872. Briefly, Drop-seq was performed on single-cell suspensions of whole lungs from 3-month-old mice (*n* = 8) and 24-month-old mice (*n* = 7). We did not modify the count matrices or annotations in the published data objects after download. The functions DotPlot() and VlnPlot() of the R package Seurat (v3.2.2)^57^ were used to visualize the normalized expression levels of *Trpml3* across the cell types. The single-cell data set in Fig. 9d and Fig. S4 has been published in Conlon et al. (2020)^56^ and was retrieved from Gene Expression Omnibus under the accession numbers GSE151674 and GSE185006. Briefly, droplet-based scRNA-seq was performed on mice which were exposed to either filtered air (control, *n* = 9) or cigarette smoke (CS, *n* = 10) for 2 (GSE185006) or 6 (GSE151674) months. The raw count matrices were filtered using the following filtering thresholds: Barcodes with more than 20% of mitochondria-encoded genes, or with less than 200 detected genes were excluded. We retained barcodes with count numbers in the range of 400 to 6000 counts per cell and genes detected in at least 3 cells. For this study, we further excluded cells from mice treated with the LTβR-Ig (*n* = 5), as the effects of LTβR-signalling were not of interest. Downstream analysis was performed using the scanpy python package (v1.8.0)^58^. Damaged droplets during scRNA-seq profiling can lead to background mRNA contamination and hamper meaningful interpretation of the data. To mitigate such effects we employed the R library SoupX^59^. We manually set the contamination fraction to 0.3 and corrected the count matrices with adjustCounts(). The expression matrices were normalized with scran’s size factor-based approach^60^ and log-transformed via scanpy’s pp.log1p() function. Variable genes were selected sample-wise, excluding known cell cycle genes. Those genes being ranked among the top 4000 in at least five samples were used as input for principal component analysis (8696 genes). Clustering was performed via scanpy’s louvain method at resolution two and cell types were manually annotated based on known marker genes. We encountered one unidentifiable cluster marked by low number of counts and the high proportion of mitochondrial transcript enriched cells, thus we marked these as low-quality cells and excluded them. The visualization was obtained with the UMAP embedding specifying the input parameters as 40 principal components and 20 nearest neighbours. The final object encompassed 27,575 genes across 26,726 cells.

### Generation and analysis of *Trpml3*^IRES-Cre/eR26-τGFP^ mice and FACS analysis

*Trpml3*^IRES-Cre/eR26-τGFP^ mice were generated as described in the Results section^61–63^. *Trpml3*^IRES-Cre/eR26-τGFP^ mice were used to analyse the expression pattern of TRPML3 in lung tissue and bronchoalveolar lavage (BAL). For cryosections female, adult mice were transcardially perfused with 4% PFA and the lung was removed. After a postfixation in 4% PFA for 2–4 h the lung was incubated in 18% sucrose solution overnight for cryoprotection. Then the lung was frozen in Tissue-Tek O.C.T. compound (4583, Sakura) and 10 µm lung cryosections were prepared followed by an immunofluorescence protocol. Various primary antibodies were used to stain different cell types of the lung. MΦ: rat anti-F4/80 (MCA497G, AbD Serootec, 1:200), B-cells: rat anti-CD45R (550286, BD Biosciences, 1:200), T-cells: rabbit anti-CD3 (C7930, Sigma, 1:200), ATII-cells: rabbit anti-SFTPC (AP13684b, Abcepta, 1:200), Cytotoxic T-cells: rabbit anti-CD8α (217344, Abcam, 1:500). Donkey antirat-Cy3 (712-165-153, Jackson ImmunoResearch, 1:500) and donkey antirabbit-Cy5 (711-175-152, Jackson ImmunoResearch, 1:500) were used as secondary antibodies. The nuclei were stained with Hoechst 33342. Pictures were taken with a Zeiss AxioScan.Z1 slide scanner running ZEN software v.2.0.0.0 and processed using the ZenBlue software 2.6 (blue edition).

For FACS analysis of BAL and lung tissue female, adult *Trpml3*^IRES-Cre/eR26-τGFP^ mice were sacrificed and BAL was isolated as described below. The lungs were perfused with 20 ml ice-cold PBS, removed and placed on petri dishes with PBS. Lung tissue was minced into pieces using scalpels and processed in a digestion buffer containing collagenase (1 mg/ml) and DNAse (0.05 mg/ml) for 30 min at 37 °C. Homogenized lungs were passed through nylon strainers (100 µm and 30 µm) to obtain a single-cell suspension. Remaining erythrocytes were lysed and resultant cells were incubated with Fc blocking antibody (TruStain FcX anti-mouse CD16/32 Antibody, Cat# 101319, Biolegend, 1:100), stained with viability dye (eBioscience Fixable Viability Dye eFluor 780, Cat# 65-0865-14, ThermoFischer) and a mixture of fluorochrome-conjugated antibodies for 20 min at 4 °C. The following antibodies were used: anti-mouse CD24 (101823, BioLegend), anti-mouse CD64 (139305, BioLegend), anti-mouse/human CD45R/B220 (103231, BioLegend), anti-mouse CD45 (103125, BioLegend), anti-mouse Ly-6G (127647, BioLegend), anti-mouse CD11c (117333, BioLegend), anti-mouse/human CD11b (101243, BioLegend), anti-mouse CD3 (100219, BioLegend) and anti-mouse MHCII (1895-09, SouthernBiotech). All antibodies were diluted 1:100. After incubation cells were washed and analysed on LSR Fortessa II (BD, Heidelberg, Germany) running BD FACSDiva software v8.0.1. Compensation was performed using UltraComp eBeads compensation beads (ThermoFischer, Cat# 01-2222-42). FACS data were analysed with FlowJo v10 (FlowJo LLC, BD) software using a sequential gating strategy to identify different cell populations^64^ (see also Results section).

Gating strategy for Fig. 1d: FSC (forward scatter) and SSC (side scatter) were used to identify lymphocytes and exclude doublets or debris. After gating for live immune cells (LD-, CD45+) only TRPML3 + cells (GFP+) were selected. In the following steps various immune cell types were excluded: T-cells (CD3e+, B220−), B-cells (CD3e-, B220+), neutrophils (Ly6G). After excluding small subsets of CD11b-/CD11c- and MHCII- cells, a big population of MΦ (CD64+ and CD24−) and a very small one of DC (CD64− and CD24+) were identified. The MHCII- subset provided monocytes/undifferentiated macrophages (CD11b + and CD64−) and NK-cells (CD11b^low^ and CD64−). DC were further classified into CD11b + DC, CD103 + DC (CD11b−), and eosinophils (MHCII- and CD11b+) were identified. The population of MΦ was divided into CD11b + interstitial macrophages (IMΦ) and CD11b- AMΦ. Gating strategy for Fig. 1f: The same sequential gating strategy as shown in Fig. 1d was applied to the first six gating steps. The resulting population was finally characterized using the markers CD11b and CD11c. The CD11c +/CD11b− population accounts for TRPML3 expressing AMΦ. The protocol was applied for 5 *Trpml3*^IRES-Cre/eR26-τGFP^ mice and 5 control mice (without GFP expression) in parallel, each. Data collected from control mice were used to set up a threshold for GFP + cells.

### Preparation of BAL

BAL was obtained from male and female mice to perform total and differential cell counts for inflammatory cell recruitment of neutrophils, macrophages and lymphocytes as well as to perform ELISA and multiplex analyses. The lungs of 16–20 weeks old *Trpml3*^*−/−*^ or WT mice were lavaged by instilling the lungs with 4 × 0.5 ml aliquots of ice-cold, sterile DPBS (Thermo-Fischer, #14190) for cytospins or with 2 × 0.5 ml aliquots of ice-cold, sterile DPBS supplemented with protease inhibitor (PI) (Roche, #04693132001) for ELISA/multiplex analysis. For cytospins, collected BAL was spun down at 400 g and cells were resuspended in 500 µl RPMI-1640 medium containing 10% FCS (both from Gibco). Total cell counts per BAL were determined in a hemocytometer or using a CASY1 TT Cell Counter & Analyser System (Roche Innovatis). Differential cell counts for neutrophils, macrophages and lymphocytes were performed using morphological criteria on May-Grünwald-Giemsa-stained cytospins (200 cells/sample). For ELISA and multiplex measurements the harvested BAL was centrifuged (1000 g, 10 min, 4 °C) to remove cells and cell debris. The obtained supernatant was distributed into aliquots, shock-frozen and stored at −80 °C until usage.

### Multiplex assays

Collected samples (see above) were stored at −80 °C and thawed on ice on the day of the experiment. For cytokine/chemokine and MMP analysis, undiluted samples were analysed using the Milliplex mouse multiplex assays MCYTOMAG-70K-11 and MMP3-MAG-79K-03 per manufacturer’s instructions. The assays were read out with a Bioplex 100 (Biorad) running Bio-Plex Manager software v4.1.1. MMP content per sample was calculated in accordance to the manufacturer’s protocol.

### Enzyme-linked immunosorbent assay (ELISA)

MMP-12, TIMP-1/2, SP-D and desmosine levels were measured by enzyme-linked immunosorbent assay. MMP-12 ELISA (SEA402Mu-96, Cloud-Clone Corp.), TIMP-1 ELISA (196265, abcam), TIMP-2 ELISA (227893, abcam) SP-D ELISA (213890, abcam), and desmosine ELISA (CSB-E14196m, Cusabio) were conducted according to the manufacturer’s protocol. BALF samples were obtained as outlined above and were analysed undiluted. O.D. absorbance at 450 nm was detected using a microplate reader (FLUOstar Omega running Reader Control software v5.50 R4, BMG LABTECH). MMP-12, TIMP-1/2, SP-D and desmosine concentrations were calculated as described in the manufacturer’s protocol.

### Whole-EE, whole-RE and whole-LE/LY manual patch-clamp experiments

For whole-EE, whole-RE and whole-LE/LY manual patch-clamp recordings, HEK-293 cells (ATCC, #CRL-1573) were treated with either a combination of wortmannin and latrunculin B (for EE enlargement), with YM201636 (for LE/LY enlargement), or after transferrin loading (Tf Alexa Fluor 555) with vacuolin (RE)^14,24^. Cells were treated with compounds at 37 °C and 5% CO_2_. YM201636 was obtained from Chemdea (CD0181), wortmannin and latrunculin B from Sigma (W1628 and L5288). Vacuolin was obtained from SantaCruz (sc-216045). Compounds were washed out before patch-clamp experimentation.

Currents were recorded using an EPC-10 patch-clamp amplifier (HEKA, Lambrecht, Germany) and PatchMaster acquisition software v2x90.4 (HEKA). Data were digitized at 40 kHz and filtered at 2.8 kHz. Fast and slow capacitive transients were cancelled by the compensation circuit of the EPC-10 amplifier. All recordings were obtained at room temperature and were analyzed using PatchMaster acquisition software (HEKA) and OriginPro 6.1 (OriginLab). Recording glass pipettes were polished and had a resistance of 4–8 MΩ. For all experiments, salt-agar bridges were used to connect the reference Ag-AgCl wire to the bath solution to minimize voltage offsets. Liquid junction potential was corrected. For the application of the lipids (A.G. Scientific) or small molecule agonists, the cytoplasmic solution was completely exchanged by a cytoplasmic solution containing agonist. The current amplitudes at −100 mV were extracted from individual ramp current recordings. Unless otherwise stated, the cytoplasmic solution contained 140 mM K-MSA, 5 mM KOH, 4 mM NaCl, 0.39 mM CaCl_2_, 1 mM EGTA and 10 mM HEPES (pH was adjusted with KOH to 7.2). The luminal solution contained 140 mM Na-MSA, 5 mM K-MSA, 2 mM Ca-MSA 2 mM, 1 mM CaCl_2_, 10 mM HEPES and 10 mM MES (pH was adjusted with methanesulfonic acid to 4.6). In all experiments, 500-ms voltage ramps from −100 to +100 mV were applied every 5 s. All statistical analysis was done using Origin8 or GraphPadPrism software.

### Whole-cell patch-clamp experiments

AMΦ isolated from male or female mice (2–6 month old) were seeded onto 12 mm coverslips and cultured for 16–40 h. Prior to the measurements, the coverslips were covered with external solution (Na^+^-Ringer solution). A glass capillary puller (Zeitz, Germany) was used to prepare recording pipettes from a borosilicate glass capillary with a resistance of 2–4 MΩ and filled with internal solution containing GTPγS and/or ML3-SA1. External solution contained 140 mM NaCl, 1 mM CaCl_2_, 2.8 mM KCl, 2 mM MgCl_2_, 10 mM HEPES NaOH, 11 mM glucose (pH was adjusted to 7.2). Internal solution contained 120 mM potassium glutamate, 8 mM NaCl, 1 mM MgCl_2_, 10 mM HEPES (pH was adjusted to 7.2). The capacity of AM was determined over time using “whole-cell” mode and an EPC-10 patch-clamp amplifier (HEKA, Lambrecht, Germany). The initial membrane capacity served as a reference value, to which the other readings were normalized. Data were analysed using the software IGOR Pro v6 (WaveMetrics). The two parameters Tau (= time until 2/3 of the maximum amplitude is reached) and Delay (= time until start of the reaction) were obtained by fitting the data with a capacitance fit function^65^. The following fit function was applied: f(x) = c_initial_ + (c_initial_ × (c_max_ − 1) × (1 − exp(−(t_delay_)/τ))^n^)^28^. To test for TRPML3 specific currents in the AMΦ plasma membrane (PM) we also applied the whole-cell patch-clamp technique. The extracellular solution contained 138 mM NaCl, 5.4 mM KCl, 2 mM CaCl_2_, 2 mM MgCl_2_, 10 mM HEPES, and 10 mM D-Glucose (311 mOsm and pH adjusted to 7.2 with NaOH). Pipette solution contained 140 mM CsCl, 10 mM HEPES, 2 mM MgCl_2_, and 1 mM EGTA (292 mOsm and pH adjusted to 7.2 with CsOH).

### Isolation and cell culture of primary peritoneal, lung tissue and AMΦ from mice

For the preparation of peritoneal and lung tissue macrophages, male or female mice (2–6 months old) were deeply anesthetized with isofluorane and killed by cervical dislocation. For harvesting peritoneal macrophages, the outer skin of the peritoneum was carefully opened and 10 ml phosphate buffer saline (PBS) were injected into the peritoneal cavity. After detaching macrophages by massaging the peritoneum, the cell suspension was collected using a syringe and a 20 G needle. Cells were pelleted and subsequently cultured in F12/DMEM supplemented with 20% FBS, 100 U penicillin/ml, and 100 µg streptomycin/ml.

Lung tissue macrophages were isolated from dissociated whole tissue by positive magnetic cell sorting (MACS) for CD11b-positive cells using the protocol for “CD11b MicroBeads, mouse” (130-049-601, Miltenyi Biotech) according to manufacturer’s instructions. Single-cell suspensions of the tissues were prepared employing the “Lung Dissociation Kit” (130-095-927; Miltenyi Biotech). Briefly, isolated tissue was rinsed in PBS, cut in 7–10 pieces and incubated in 2.4 ml 1x buffer S containing enzyme A and enzyme D for 45 min at 37 °C. Afterwards, cells were passed through a 100 µm nylon mesh followed by one more separation through a 30 µm nylon mesh. The cell suspension was centrifuged and resuspended in red cell lysis buffer (Sigma, R7757) to remove erythrocytes. Following a further centrifugation step, cells were recollected with MicroBeads conjugated to monoclonal rat anti-mouse CD11b antibody and incubated for 15 min at 4 °C. CD11b-positive cells were sorted with MS MiniMACS columns and the eluted fraction was seeded onto Poly-L-Lysine coated coverslips and maintained in F12/DMEM containing 20% FBS, 100 U penicillin/ml, and 100 µg streptomycin/ml.

For isolation of AMΦ male or female mice (2–6 months old) were deeply anesthetized by intraperitoneal injection of ketamine-xylazine and killed through exsanguination. The diaphragm of the lung was opened through a small cut leading to a collapse of the lungs. After removing the tissue from the neck to expose the trachea, a small cut was made between the cartilage rings to open the trachea. A cannula (Introcan-W, 20 G x 1¼, B. Braun Melsungen AG) was carefully inserted into the trachea and fixed by a suture placed around the cannulated trachea. Using 1 ml syringes the lungs were flushed with ~0,8 ml of ice-cold DPBS for at least seven times to have a high yield of cells. Each time after infusing the DPBS into the lungs, the fluid was withdrawn carefully into the syringe and collected in a tube kept on ice. Finally, the lavage was centrifuged at 1000 g, 4 °C for 10 min and the cell pellet was cultured in RPMI containing 10% FBS, 100 U penicillin/ml, and 100 µg streptomycin/ml.

### Genotyping and RT-qPCR

*Trpml1*^*−/−*^ mice were obtained from Dr. Susan Slaugenhaupt (Harvard University, Boston, USA)^66^. For genotyping of *Trpml1*^*−/−*^ mice the following forward and reverse primers were used: 5′-tgaggagagccaagctcatt-3′ (sense), 5′-tcatcttcctgcctccatct-3′ (antisense) and 5′-tggctggacgtaaactcctc-3′ (antisense), expected bands 400 bp (WT), 200 bp (KO); cycling conditions: annealing temperature 58 °C, 35 cycles. For genotyping of *Trpml3*^*−/−*^ (*Mcoln3*^*tm1.2Hels*^) and WT mice two primer pairs were used: 5′-gaacacactgactacccccaa-3′ (sense) and 5′-tacagttttacagatgtgtttgag-3′ (antisense), expected bands: 309 bp (WT), no band (KO); 5′-gaacacactgactacccccaa-3′ (sense), and 5′-agaggttcactagaacgaagttcctattcc-3′ (antisense), expected bands: no band (WT), 374 bp (KO); cycling conditions: 35 cycles, annealing temperature 59 °C for both. *Trpml3*^*−/−*^ mice (*Mcoln3*^*tm1.1Jga*^) were obtained from Dr. Jaime García-Añoveros^13^. For genotyping of *Trpml3*^*−/−*^ mice the following forward and reverse primers were used: 5′-ctgtgagacctcttaacaactct-3′ (sense), 5′-gtggagccttgactgtctag-3′ (antisense) and 5′-ggcaagagctg aggatatctt-3′ (antisense), expected bands: 263 bp (WT), 443 bp (KO); cycling conditions: annealing temperature 51 °C, 35 cycles.

Total RNA was prepared from cultured primary macrophages using RNeasy Plus Mini Kit (Qiagen) according to the manufacturer’s protocol. cDNA was synthesized from total RNA with RevertAid First Strand cDNA Synthesis Kit (Thermo Scientific) utilizing both random hexamer primer and oligo(dT)_18_-primer. qPCR was performed on a StepOne Plus Real-time PCR system (Applied Biosystems, StepOne software v2.3) using SYBR Select Master Mix (Applied Biosystems) or on a Light Cycler 480 Instrument (Roche, Light Cycler 480 software v1.5.1) using LightCycler 480 SYBR Green I Master Mix (Roche). Reactions were carried out in duplicate or triplicate under conditions according to manufacturer’s recommendations. The following forward and reverse primers were used for TRPML1 (NM_053177), TRPML2 (NM_026656), TRPML3 (NM_134160), HPRT (NM_013556), MMP-12 (NM_008605), GAPDH (NM_008084) and ACTB (NM_007393): 5′-gccttgggccaatggatca-3′ (sense), 5′-cccttggatcaatgtcaaaggta-3′ (antisense) (TRPML1), 5′-aatttggggtcacgtcatgc-3′ (sense), 5′-agaatcgagagacgccatcg-3′ (antisense) (TRPML2), 5′-gagttacctggtgtggctgt-3′ (sense), 5′-tgctggtagtgcttaattgtttcg-3′ (antisense) (TRPML3), 5′-gctcgagatgtcatgaaggagat-3′ (sense), 5′-aaagaacttatagccccccttga-3′ (antisense) (HPRT), 5′-ctgcctcatcaaaatgtgcatc-3′ (sense), 5′-atttggagctcacggagactt-3′ (antisense) (MMP-12), 5′-ccaccaccctgttgctgtag-3′ (sense), 5′-ctcccactcttccaccttcg-3′ (antisense) (GAPDH), and 5′-cacagcctggatggctacgt-3′ (sense), 5′-ctaaggccaaccgtgaaaagat-3′ (antisense) (ACTB). Primer efficiencies were between 1.9 and 2.1. Nontemplate controls were included to ensure the specificity of the primer pairs. Product specificity and amplicon size were controlled by sequencing and gel analysis of the qPCR products. Relative expression of target gene levels was determined by normalization against HPRT, GAPDH, or ACTB levels.

### Endocytosis experiments

Endocytosis experiments were performed using dextran, Alexa Fluor 568; 10,000 MW, anionic, fixable (D22912, Molecular Probes). AMΦ isolated from female or male mice (WT vs. *Trpml3*^−/−^, 2–6 months old) were seeded overnight in phenolred-free DMEM supplemented with 10% FBS, 100 U penicillin/ml, and 100 μg streptomycin/ml. For the assay, the cells were pulsed with fluorescently labelled dextran (50 µg/ml) in serum-free DMEM (37 °C) for different time periods (5–30 min). After removing the dextran-containing media, cells were washed with DPBS and fixed with 4% paraformaldehyde (PFA) for 10 min followed by a DAPI staining. Cells were imaged using a Zeiss LSM880 with 40x magnification and running ZEN software v2.3 SP1. For the analysis ImageJ software v1.52p was used to measure the fluorescence intensity in the macrophages excluding the nucleus. The relative increase of fluorescence intensity over time was determined by normalization to untreated control cells.

### Detection of MMP-8 and MMP-12 levels in supernatant from cultured AMΦ

AMΦ were isolated from female and male WT and *Trpml3*^*−/−*^ mice. All WT AMΦ were pooled together, as well as all *Trpml3*^*-/-*^ AMΦ, to obtain the highest possible cell count per genotype. Cells were then seeded in phenolred-free RPMI supplemented with 10% FBS, 100 U penicillin/ml, and 100 μg streptomycin/ml in wells of a 96-well plate, 100,000 cells per well. After one day the cells were washed with medium to remove the non-adherent cells before refreshing the media with 200 µl phenolred-free RPMI containing PI and supplemented with 10% FBS, 100 U penicillin/ml, and 100 μg streptomycin/ml. Cells were then cultured for 72 h. To inhibit endocytosis in WT AM several endocytosis blockers were added to the media. Endocytosis blockers and their final concentrations were: Dynasore (Dyn) 50 μg/ml, methyl-ß-cyclodextrin (MBCD) 2.5 mg/ml and 5-(N-ethyl-N-isopropyl)amiloride (EIPA) 150 μM (all from Sigma). For basal condition (= without endocytosis inhibition) the appropriate volume of media containing DMSO was added. For every condition a blank control was prepared in an extra well, without cells, only consisting of medium + endocytosis inhibitor/DMSO. After 4 h of incubation the SN from all wells were collected into tubes on ice. Samples were centrifuged at 11000 g for 10 min at 4 °C and shock frozen in liquid nitrogen before transferring into -80 °C freezer until usage. MMP-8 and MMP-12 concentrations in the SN were measured by ELISA (ab206982 and ab213878, Abcam) according to the manufacturer’s protocol. Samples were analysed 1:1 diluted. O.D. absorbance at 450 nm was detected using a microplate reader (FLUOstar Omega running Reader Control software v5.50 R4, BMG LABTECH). MMP-8 and MMP-12 concentrations were calculated as described in the manufacturer’s protocol.

### Transferrin trafficking experiments

AMΦ isolated from female or male mice (WT vs. *Trpml3*^−/−^, 2–6 months old) were seeded overnight in phenolred-free DMEM supplemented with 10% FBS, 100 U penicillin/ml, and 100 μg streptomycin/ml. Cells were incubated for 10 min at 4 °C on ice. Then, cells were pulsed for 20 min at 37 °C at 5% CO_2_ with transferrin from human serum, Alexa Fluor 488-conjugated (T13342, ThermoFisher) at the concentration of 20 µg/ml in serum-free DMEM. The reaction was quenched by washing the cells three times with 0.1 M glycine-PBS. Recycling kinetics were analysed by chasing for 5, 10, 15, and 20 min in complete media plus 20 µg/ml unconjugated transferrin (T0665, Sigma). After fixation with 4% PFA the nuclei were stained with DAPI. Images were acquired using a Zeiss LSM880 with 40x magnification and running ZEN software v2.3 SP1. For the analysis, ImageJ software v1.52p was used to measure the fluorescence intensity in the macrophages excluding the nucleus. The relative decrease of fluorescence intensity over time was determined by normalization to 0 min timepoint. For colocalization experiments of early endosomes with Tf+ vesicles, cells were stained for the early endosomal marker EEA1 (C45B10, Cell signaling, 1:100) after the 20 min Tf-pulse and PFA fixation. Colocalized fractions were analysed using ImageJ.

### Lysosomal exocytosis assay (Hexosaminidase assay)

AMΦ isolated from female or male mice (WT vs. *Trpml3*^−/−^, 2–6 months old) were seeded overnight in wells of a 96 well plate (60 000 cells per well). Cells were treated with DMSO (60 min), 4 µM ionomycin calcium salt (10 min) (I0634, Sigma) or 10 µm ML3-SA1 (60 min) in serum-free and phenolred-free DMEM medium. After treatment, supernatants were collected and kept on ice. Cells were lysed with lysis buffer (25 mM HEPES, 150 mM NaCl, 0.5% Triton-X) for 30 min on ice. Supernatants and lysates were centrifuged and incubated with sodium citrate buffer (pH 4.5) and 4-methylumbelliferyl N-acetyl- ß-D-glucosaminide (M2133, Sigma, 1 mM final concentration) for 30 min at 37 °C. The reaction was stopped by adding glycine buffer to the samples. The turnover of hexosaminidase substrate (MUF) was detected as fluorescence (excitation: 365 nm; emission: 450 nm) using a plate reader (Spectramax ID3 running SoftMax Pro software v6, Molecular Devices). The increase in substrate turnover was analysed as fluorescence increase in supernatants relative to the total turnover from supernatants and lysates.

### Lysosomal exocytosis assay (LAMP1 translocation assay)

AMΦ isolated from female or male mice (WT vs. *Trpml3*^−/−^, 2–6 months old) were seeded on 8-well plates (Ibidi) and cultured overnight. After one wash with PBS cells were treated with DMSO (for 120 min), 4 µM ionomycin (for 10 min), and 30 µM ML3-SA1 (for 60 and 120 min, each) in Minimum Essential Media (MEM) supplemented with 10 mM HEPES. Then cells were incubated with an anti-LAMP1 antibody (1:200, sc-19992, SantaCruz) in MEM supplemented with 10 mM HEPES and 1% BSA for 20 min on ice. After fixation with PFA (28906, Thermo Fisher) for 20 min cells were incubated with Alexa Fluor 488-conjugated secondary antibody (1:400, Thermo Fisher) for 1 hour in PBS containing 1% BSA. Nuclei were stained with DAPI. Confocal images were acquired using LSM880 microscope (Zeiss) with 40x magnification and running ZEN software v2.3 SP1.

### Western Blotting

AMΦ isolated from female or male mice (WT vs. *Trpml3*^−/−^, 2–6 months old) were isolated and cell pellets were resuspended in lysis buffer (10 mM TRIS HCl pH 8 and 0.2% SDS) supplemented with proteinases and phosphatases inhibitor (Sigma). Total cell lysis was completed by ultrasonication. Protein concentration was determined by the Bradford method. SDS-polyacrylamide gel electrophoresis (PAGE), immunoblotting, protein visualization, membrane developing using Odyssey FC Imaging System (LI-COR) running ImageStudio software v1.0.19 and protein quantification were performed according to established protocols^67^. Sample processing controls for quantitative comparison were run on the same blots as the samples, but the blots were cut before incubation with antibodies to detect the respective protein bands. The following antibodies were used: ß-actin (Cell Signaling, 4970, 1:100 or SantaCruz, 47778, 1:1000), transferrin receptor (ThermoFisher, 13-6800, 1:500), LC3B (Novus Biologicals, 100-2220, 1:1000), Phospho-NF-κB p65 (Ser536) (Cell Signaling, 3033, 1:1000), NF-κB p65 (Cell Signaling, 6956, 1:1000), Phospho-NF-κB p105 (Ser932) (Cell Signaling, 4806, 1:1000) and NF-κB1 p105/p50 (Cell Signaling, 13586, 1:1000). Uncropped scans of all blots are supplied with the Source Data file.

### LDH-Cytotoxicity Assay

AMΦ were isolated from female or male WT and *Trpml3*^*−/−*^ mice (2–6 months old). Cells were then seeded in phenolred-free RPMI supplemented with 10% FBS, 100 U penicillin/ml, and 100 μg streptomycin/ml in wells of a 96-well plate, 60,000 cells per well. After one day the cells were washed with a fresh medium to remove the non-adherent cells. Cells were then incubated overnight with 100 µl phenolred-free RPMI containing either 30 µM ML3-SA1 or DMSO and supplemented with 10% FBS, 100 U penicillin/ml, and 100 μg streptomycin/ml. On the next day, LDH levels were measured in the cell culture medium as a marker for cytotoxicity according to the manufacturer’s protocol of the LDH Assay Kit (ab6593).

### Lipidomics

Lipids were extracted from 25 μl BALF using methyl-tert.-butyl ether^68^. Lipid identification and quantification were carried out using the shotgun lipidomics assistant^69^, which is essentially an extended open access version of the Lipidyzer platform^70^.

### Generation of bone marrow-derived macrophages (BMDM) polarization and differentiation

Bone marrow was flushed from femurs and tibias of male *Trpml3*^−/−^ mice and WT littermate controls (5 months old) with RPMI-1640 medium. The suspension was passed through 40 µm filters (Mitleny biotec), counted and resuspended in RPMI-1640 medium (Gibco, Life Technologies) supplemented with 5% fetal bovine serum (Gibco, Life Technologies), 50 µM β-mercaptoethanol and 100 U/ml penicillin and streptomycin (both Sigma-Aldrich). 2 × 10^6^ cells/ml were plated in 24 well plates and 20 ng/mL of murine recombinant M-CSF (ImmunoTools) were added to the medium. Cells were maintained at 37 °C, 5% CO2 for 7days changing medium every 3rd day and carefully discarding non-adherent cells. On day 7, a fresh medium without M-CSF was added and left overnight. In order to obtain M0 cells, adherent cells were harvested the next day, counted and seeded at a density of 1 × 10^6^ cells/ml in 24 well plats and cultured for 24 h in a fresh medium. For M1 differentiation, cells were cultured in a medium containing 1 μg/ml LPS (Sigma-Aldrich) and 20 ng/ml recombinant murine IFNγ (ImmunoTools) and for M2 medium containing 20 ng/ml recombinant murine IL-4 (ImmunoTools). FACS analysis were performed on freshly harvested bone marrow as well as day 7 bone marrow-derived macrophages. Single-cell suspensions were first blocked with purified anti-mouse CD16/CD32 (clone 93, eBIoscience, ThermoFischer Scientific) before incubating for 30 min on ice with the following cocktail; VioGreen-conjugated anti-CD45 (clone: 30F11, Miltenyi Biotec), PerCP-Vio700-conjugated anti F4/80 (clone: REA126, Miltenyi Biotec), PE-conjugated anti-CD11b (clone: M1/70.15.11.5, Miltenyi Biotec) and APC-conjugated anti-CD11c (clone: N418, Miltenyi Biotec). All antibodies were diluted 1:100. Cells were analyzed on a BD FACSCanto II flow cytometer (BD Biosciences) with BD FACSDiva v6.1.3 software. In addition, total RNA was isolated using peqGOLD Kit (Peqlab), cDNA was synthesized from 1 μg total RNA using Random Hexamers and MuLV Reverse Transcriptase (Applied Biosystems). mRNA expression was analyzed using Platinum SYBR Green qPCR SuperMix (Applied Biosystems) on a StepOnePlusTM 96 well Real-Time PCR System (Applied Biosystems). Primers were designed using Primer-BLAST software: *Hprt1* fw: AGC TAC TGT AAT GAT CAG TCA ACG, rev: AGA GGT CCT TTT CAC CAG CA; *Arg1* fw: GGA ACC CAG AGA GAG CAT GA, rev: TTT TTC CAG CAG ACC AGC TT; *Fizz1* fw: TGC CAA TCC AGC TAA CTA TCC C, rev: ACG AGT AAG CAC AGG CAG TT; *Il1b* fw: AGT TGA CGG ACC CCA AAA GAT, rev: GGA CAG CCC AGG TCA AAG G; *Inos* fw: CGG CAA ACA TGA CTT CAG GC, rev: GCA CAT CAA AGC GGC CAT AG.

### MCOLN3 expression in human lung and lavage published data sets

Series matrix files from the NCBI GEO database for GSE27597, GSE8823 and GSE2125 were downloaded. Gene expression of MCOLN3 in all lung tissue samples from GSE27597 (*n* = 16 lung samples from two smokers; *n* = 48 lung samples from six smokers with COPD; expression profiling by array) was calculated relative to the mean expression value across all the healthy samples and reported as fold change. MCOLN3 in all samples from GSE8823 (alveolar macrophages obtained by bronchoalveolar lavage from *n* = 11 nonsmokers and *n* = 13 smokers; expression profiling by array) and GSE2125 (alveolar macrophages obtained by bronchoalveolar lavage from *n* = 15 nonsmokers and *n* = 15 smokers; expression profiling by array) was calculated relative to the mean expression across all the nonsmokers samples from the respective data set and reported as fold change. Statistical significance was determined using a two-tailed Mann–Whitney test.

### Reporting summary

Further information on research design is available in the Nature Research Reporting Summary linked to this article.

## Supplementary information

Supplementary Information

Reporting Summary

## Data Availability

All data supporting the findings from this study are available within the manuscript and its supplementary information. The scRNA-seq data used in this study were not generated in this manuscript and are available in the Gene Expression Omnibus database under the accession codes GSE124872, GSE151674 and GSE185006. The human array data sets were not generated in this manuscript and are available in the Gene Expression Omnibus database under the accession codes GSE27597, GSE8823, and GSE2125. Source data are provided with this paper.

## Source data

Source data
