# Supplementary material for: Lung emphysema and impaired macrophage elastase clearance in mucolipin 3 deficient mice
Source: Nat Commun. 2022 Jan 14;13:318. doi: 10.1038/s41467-021-27860-x (PMC8760276; doi:10.1038/s41467-021-27860-x)
Supplement: Supplementary file 1 — Supplementary Information [file 41467_2021_27860_MOESM1_ESM.pdf]

# Supplementary information

# Lung emphysema and impaired macrophage elastase clearance in mucolipin 3 deficient mice

Barbara Spix[1], Elisabeth S. Butz[2,3], Cheng-Chang Chen[2,4], Anna Scotto Rosato[1], Rachel Tang[1], Aicha Jeridi[5], Veronika Kudrina[1], Eva Plesch[2], Philipp Wartenberg[6], Elisabeth Arlt[1], Daria Briukhovetska[7], Meshal Ansari[5], Gizem Günes Günsel[5], Thomas M. Conlon[5], Amanda Wyatt[6], Sandra Wetzel[8], Daniel Teupser[9], Lesca M. Holdt[9], Fabien Ectors[10], Ingrid Boekhoff[1], Ulrich Boehm[6], Jaime García-Añoveros[11], Paul Saftig[8], Martin Giera[12], Sebastian Kobold[7,13], Herbert B. Schiller[5], Susanna Zierler[1,14], Thomas Gudermann[1,15], Christian Wahl-Schott[3], Franz Bracher[2], Ali Önder Yildirim[5]*, Martin Biel[2]*, Christian Grimm[1]*

### Supplementary Note 1

**(±)-*cis*-3-Mesityl-3a,5,6,7a-tetrahydro-4*H*-pyrano[3,2-*d*]isoxazole (EVP-77 = ML3-SA1)**

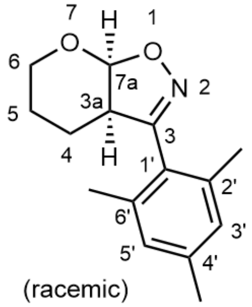


$C_{15}H_{19}NO_2$, $M_r$ = 245.32

A solution of mesitonitrile oxide (406 mg, 2.52 mmol) and 3,4-dihydro-2*H*-pyran (375 mg, 4.46 mmol) in ethyl acetate (3 mL) was stirred for 30 hours at ambient temperature. The volatiles were removed under reduced pressure and the residue was purified by flash column chromatography (hexanes/ethyl acetate 3:1) to give colorless crystals (294 mg, 48 %). mp: 92 °C; $^1$H-NMR (500 MHz, $CDCl_3$) δ (ppm) = 6.91 (s, 2H, 3'-H, 5'-H), 5.72 (d, $J_1$ = 5.9 Hz, 1H, 7a-H), 3.91 (dtd, $J_1$ = 11.6 Hz, $J_2$ = 4.4 Hz, $J_3$ = 1.4 Hz, 1H, 6-H), 3.59 (ddd, $J_1$ = 6.7 Hz, $J_2$ = 6.0 Hz, $J_3$ = 2.7 Hz, 1H, 3a-H), 3.52 (ddd, $J_1$ = 11.7 Hz, $J_2$ = 9.2 Hz, $J_3$ = 4.1 Hz, 1H, 6-H), 2.30 (s, 3H, 4'-$CH_3$), 2.29 (s, 6H, 2'-$CH_3$, 6'-$CH_3$),

1.92 – 1.83 (m, 1H, 4-H), 1.71 – 1.62 (m, 1H, 4-H), 1.51 – 1.40 (m, 2H, 5-H). $^{13}$C-NMR (101 MHz, $CDCl_3$) δ (ppm) = 161.6 (C-3), 139.1 (C-4'), 137.3 (C-2', C-6'), 129.2 (C-3', C-5'), 124.7 (C-1'), 99.9 (C-7a), 62.1 (C-6), 48.4 (C-3a), 21.7 (C-5), 21.2 (4'-$CH_3$), 20.7 (2'-$CH_3$, 6'-$CH_3$), 19.4 (C-4). IR (KBr) ṽ [$cm^{-1}$] = 3434, 2985, 2950, 2935, 2889, 2874, 2863, 1611, 1447, 1435, 1382, 1324, 1258, 1217, 1117, 1106, 1057, 1000, 964, 905, 855, 819, 802, 734. MS (EI) *m/z* (%) = 245 (64) $[M^{\cdot}]^+$, 172 (45), 161 (100). HRMS (EI) 245.1419 (calculated for $C_{15}H_{19}NO_2$: 245.1416). Purity (HPLC) 99 % (λ = 210 nm), > 99 % (λ = 254 nm).

**(±)-4,5,6,7-Tetrachloro-2-[2-oxo-2-(2,2,4-trimethyl-3,4-dihydrochinolin-1(2*H*)-yl)ethyl]isoindolin-1,3-dione (EVP-169 = ML1-SA1)**

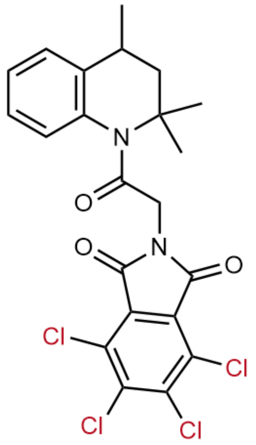

(racemic)

$C_{22}H_{18}Cl_4N_2O_3$, $M_r$ = 500.20

A mixture of racemic 1-(chloroacetyl)-2,2,4-trimethyl-1,2,3,4-tetrahydroisoquinoline (104 mg, 0.413 mmol), tetrachlorophthalimide ( 244 mg, 0.788 mmol), sodium iodide (119 mg, 0.794 mmol) and molecular sieves (4 Å; 3.0 g) was stirred in dry dioxane (4 mL) under a nitrogen atmosphere. A solution of LiHMDS in toluene (1 M; 0.500 mL, 0.500 mmol) was added slowly. After stirring the reaction mixture for 48 hours at 105 °C and cooling it down to room temperature, the molecular sieves was removed by filtration and washed with dichloromethane (20 mL). The combined organic layers were washed with brine (3 x 20 mL) and dried over sodium sulfate. The volatiles were removed under reduced pressure and the residue was purified by flash column chromatography (hexanes/ethyl acetate 9:1) to give yellow crystals (111 mg, 54 %). mp: 189 °C; $^1$H-NMR (500 MHz, $CDCl_3$) δ (ppm) = 7.25 – 7.17 (m, 4H, 5''-H, 6''-H, 7''-H, 8''-H), 4.81 (d, *J* = 16.1 Hz, 1H, 1'-H), 4.05 (d, *J* = 16.0 Hz, 1H, 1'-H), 2.87 (dqd, $J_1$ = 13.5 Hz, $J_2$= 6.7 Hz, $J_3$ = 2.6 Hz, 1H, 4''-H), 1.86 (dd, $J_1$ = 12.8 Hz, $J_2$ = 2.7 Hz, 1H, 3''-H), 1.66 (s, 3H, 2''-$CH_3$), 1.44 (s, 3H, 2''-$CH_3$), 1.34 (d, *J* = 6.8 Hz, 3H, 4''-$CH_3$), 1.22 (dd, $J_1$ = 13.5 Hz, $J_2$ =12.7 Hz, 1H, 3''-H). $^{13}$C-NMR (126 MHz, $CDCl_3$) δ (ppm) = 166.3

(C-2'), 163.3 (C-1, C-3), 141.8 (C-4a''), 140.1 (C-5, C-6), 137.4 (C-8a''), 129.8 (C-4, C-7), 127.9 (C-3a, C-7a), 126.6 (C-7''), 126.1 (C-6''), 125.1 (C-8''), 123.6 (C-5''), 60.5 (C-2''), 51.7 (C-3''), 42.9 (C-1'), 29.3 (C-4''), 27.9 (2''-$CH_3$), 25.8 (2''-$CH_3$), 16.9 (4''-$CH_3$). IR (ATR) ṽ [$cm^{-1}$] = 2958, 2923, 2879, 1785, 1723, 1666, 1425, 1397, 1311, 1295, 1130, 761, 740. MS (EI) m/z (%) = 504 (2) [($^{35}Cl$, $^{37}Cl_3$) $M^{\bullet}$]$^+$, 502 (8) [($^{35}Cl_2$, $^{37}Cl_2$) $M^{\bullet}$]$^+$, 500 (14) [($^{35}Cl_3$, $^{37}Cl$) $M^{\bullet}$]$^+$, 498 (12) [($^{35}Cl_4$) $M^{\bullet}$]$^+$, 298 (24), 202 (24), 160 (80), 146 (100). HRMS (EI) 498.0065 (calculated for $C_{22}H_{18}{}^{35}Cl_4N_2O_3$: 498.0072). Purity (HPLC) > 99 % (λ = 210 nm), > 99 % (λ = 254 nm).

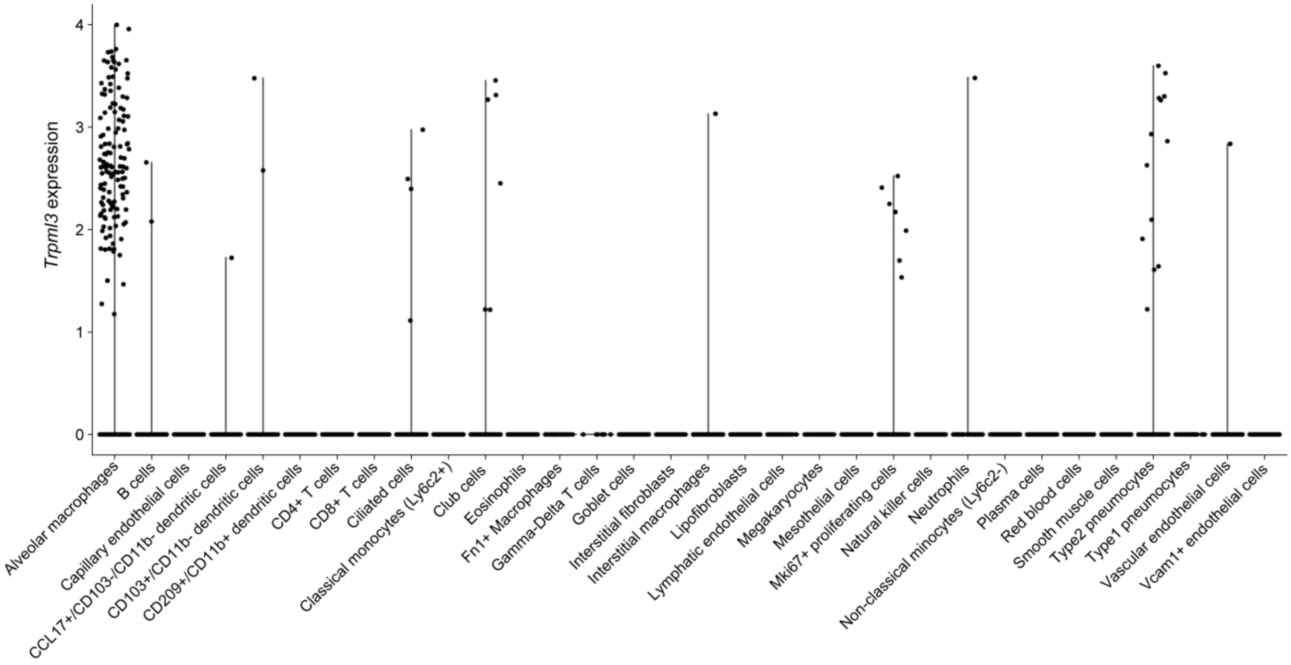


## Supplementary Figure S1

**Data of Fig. 1h shown as violin plot.** Data can be obtained from Gene Expression Omnibus under the accession code GSE124872.

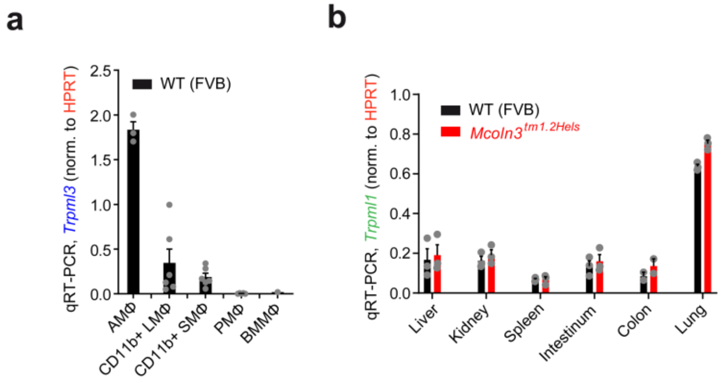


## Supplementary Figure S2

***Trpml1* and *Trpml3* expression in murine macrophages and organs.** (**a-b**) qRT-PCR results showing levels of *Trpml1* and *Trpml3* mRNA in different macrophage populations (a) and different murine tissues (b). AMΦ = alveolar macrophages, LMΦ = lung tissue macrophages, SMΦ = splenic macrophages, PMΦ = peritoneal macrophages, BMMΦ = bone marrow macrophages. Shown are normalized mean values ± SEM. Each dot represents one biologically independent experiment. Source data are provided as a Source Data file.

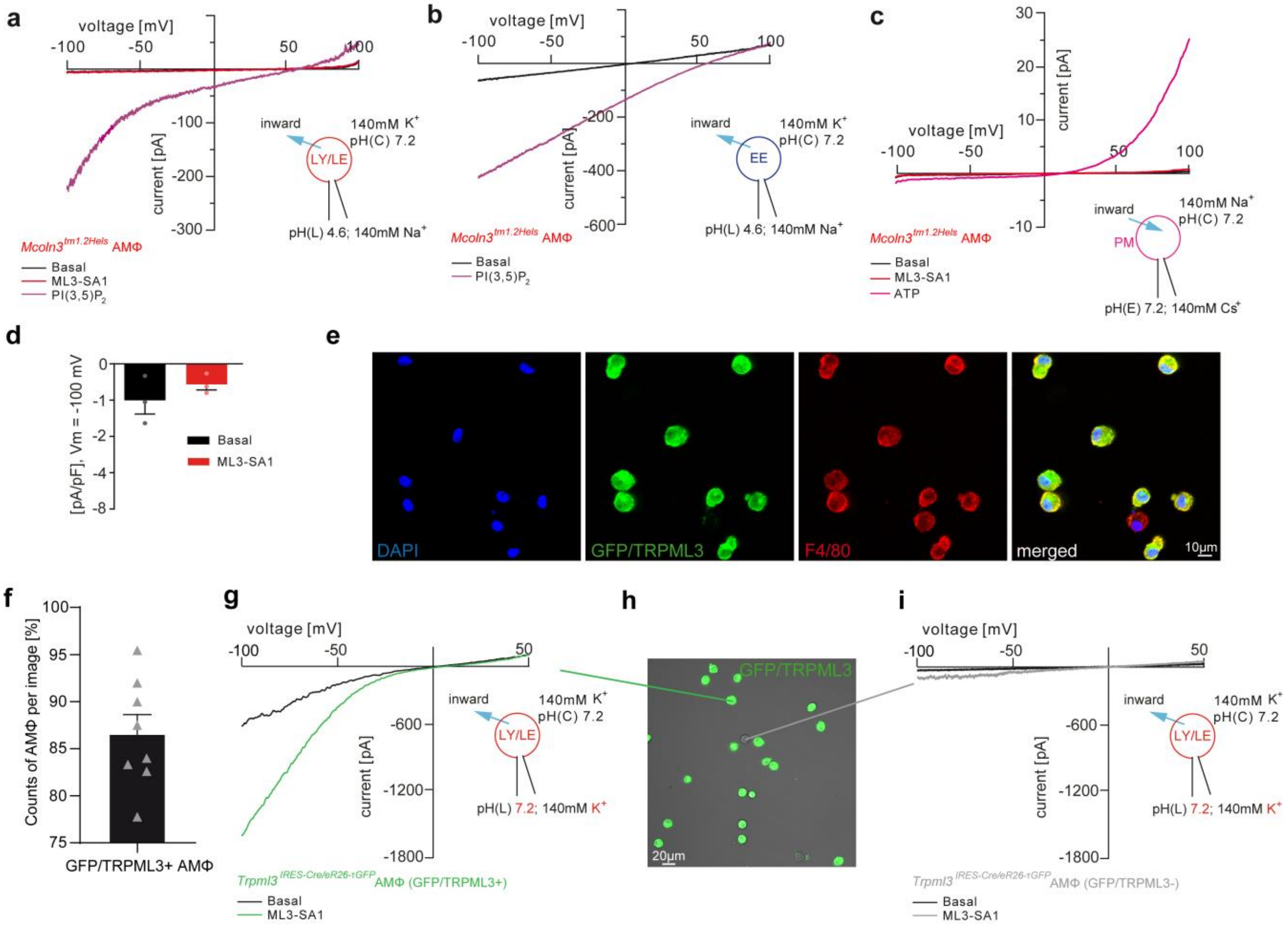


## Supplementary Figure S3

**Positive control currents in EE and LE/LY isolated from *Trpml3*$^{-/-}$ murine AMΦ, PM currents and currents from *Trpml3*$^{IRES\text{-}Cre/eR26\text{-}\tau GFP}$ mouse AMΦ.** (**a-b**) Representative currents from YM201636-enlarged LE/LY (a) and Wort./Lat.B-enlarged EE (b) isolated from murine *Trpml3*$^{-/-}$ primary AMΦ, elicited by an application of 1 µM PI(3,5)$P_2$, respectively. In a LE/LY mixed current of TRPML1 and TPCs was detectable while in EE only TPC currents remained detectable. L = luminal, C = cytosolic (**c**) Representative plasma membrane (PM) whole-cell currents elicited with 30 µM ML3-SA1 or 1 mM ATP (the latter was used as positive control). No significant TRPML3 currents were detectable. E = extracellular, C = cytosolic (**d**) Statistics of experiments as shown in c. Each dot represents one biologically independent experiment. Data are mean ± SEM. (**e**) Immunofluorescence experiments (representative images) of AMΦ purified from *Trpml3*$^{IRES\text{-}Cre/eR26\text{-}\tau GFP}$ reporter mice and co-stained with F4/80 as marker for macrophages and DAPI to visualize nuclei. (**f**) Quantification of GFP/TRPML3+ AMΦ in samples as shown in e. Each dot represents one biologically independent sample. Data are mean ± SEM. (**g**) Representative endolysosomal currents elicited with 10 µM ML3-SA1 in YM201636-enlarged LE/LY isolated from TRPML3/GFP+ AMΦ purified from *Trpml3*$^{IRES\text{-}Cre/eR26\text{-}\tau GFP}$ reporter mice. (**h**) Representative lower magnification image showing GFP- and GFP/TRPML3 expressing AMΦ purified from *Trpml3*$^{IRES\text{-}Cre/eR26\text{-}\tau GFP}$ reporter mice used for endolysosomal patch-clamp experiments shown in g and i. (**i**) Representative endolysosomal currents elicited with 20 µM ML3-SA1 in YM201636-enlarged LE/LY isolated from GFP- AMΦ purified from *Trpml3*$^{IRES\text{-}Cre/eR26\text{-}\tau GFP}$ reporter mice. Source data are provided as a Source Data file.

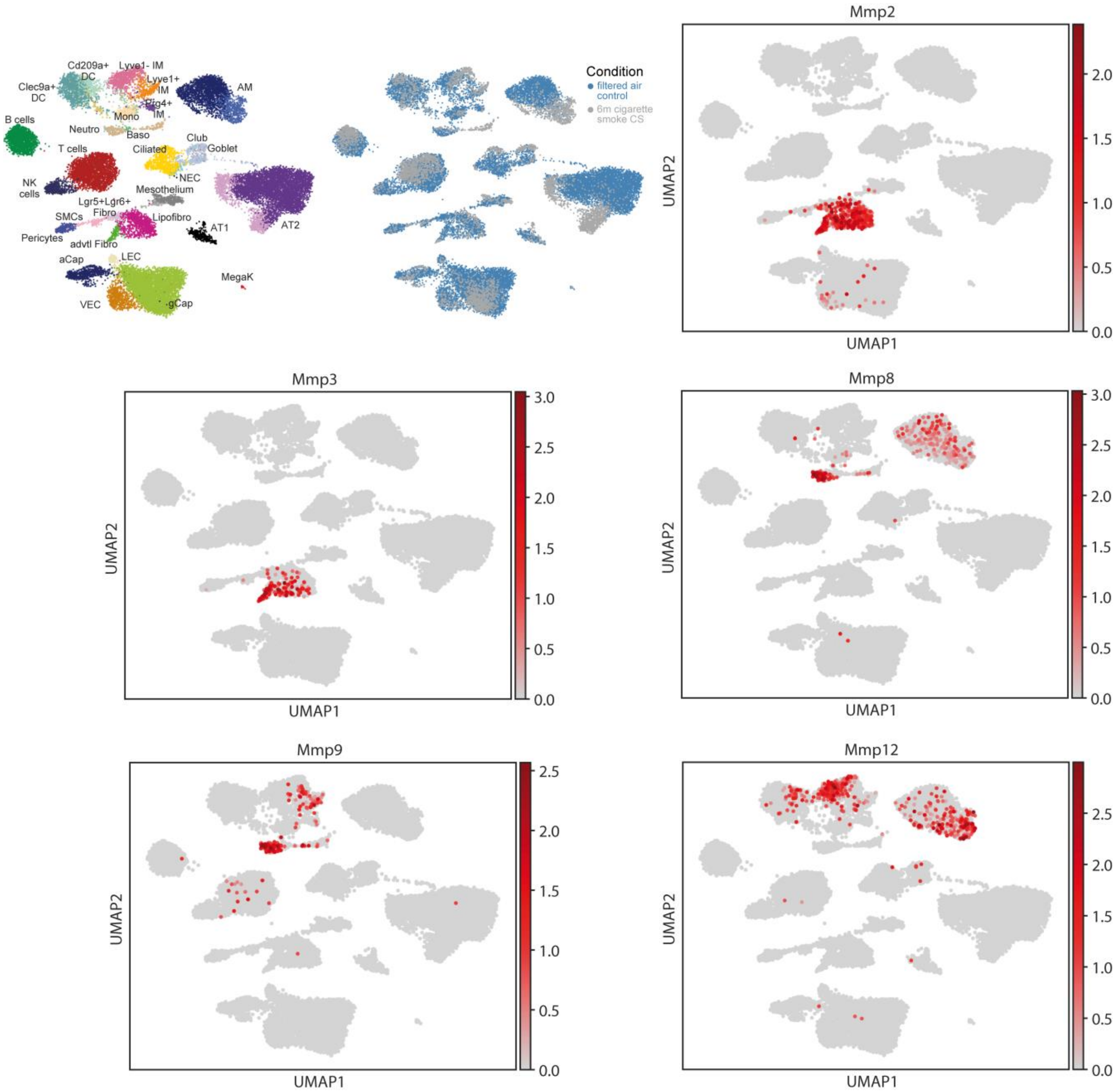


## Supplementary Figure S4

**Expression of selected MMPs in the lung.** The UMAP shows Drop-seq single-cell data from murine whole lungs coloured by cell type and condition. Mice were exposed to either filtered air (control, n = 9) or cigarette smoke (CS, n = 5) for 2 or 6 months. The normalized expression levels of indicated genes are overlaid on the UMAP embedding. Data can be obtained from Gene Expression Omnibus under the accession codes GSE185006 for 2 months and GSE151674 for 6 months.

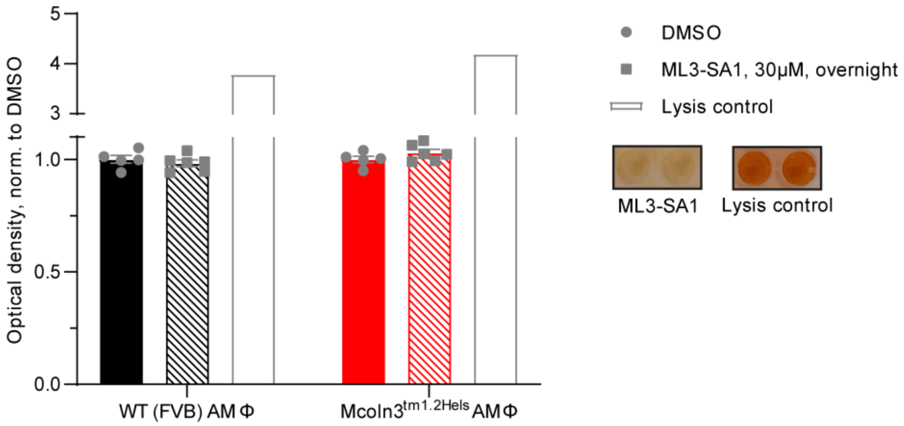


**Supplementary Figure S5**

**Cytotoxicity of ML3-SA1.** Cytotoxicity of ML3-SA1 was assessed by measuring the lactate dehydrogenase (LDH) levels in cell culture medium from cultured AMΦ isolated from WT and *Trpml3*$^{-/-}$ mice. Cells were either treated with 30 µM ML3-SA1 overnight or DMSO before the assay was performed. A lysis control (supplied with the kit) was used as a positive control. ML3-SA1 did not show a cytotoxic effect on the AMΦ. Each dot represents one biologically independent sample. Data are mean ± SEM. Source data are provided as a Source Data file.

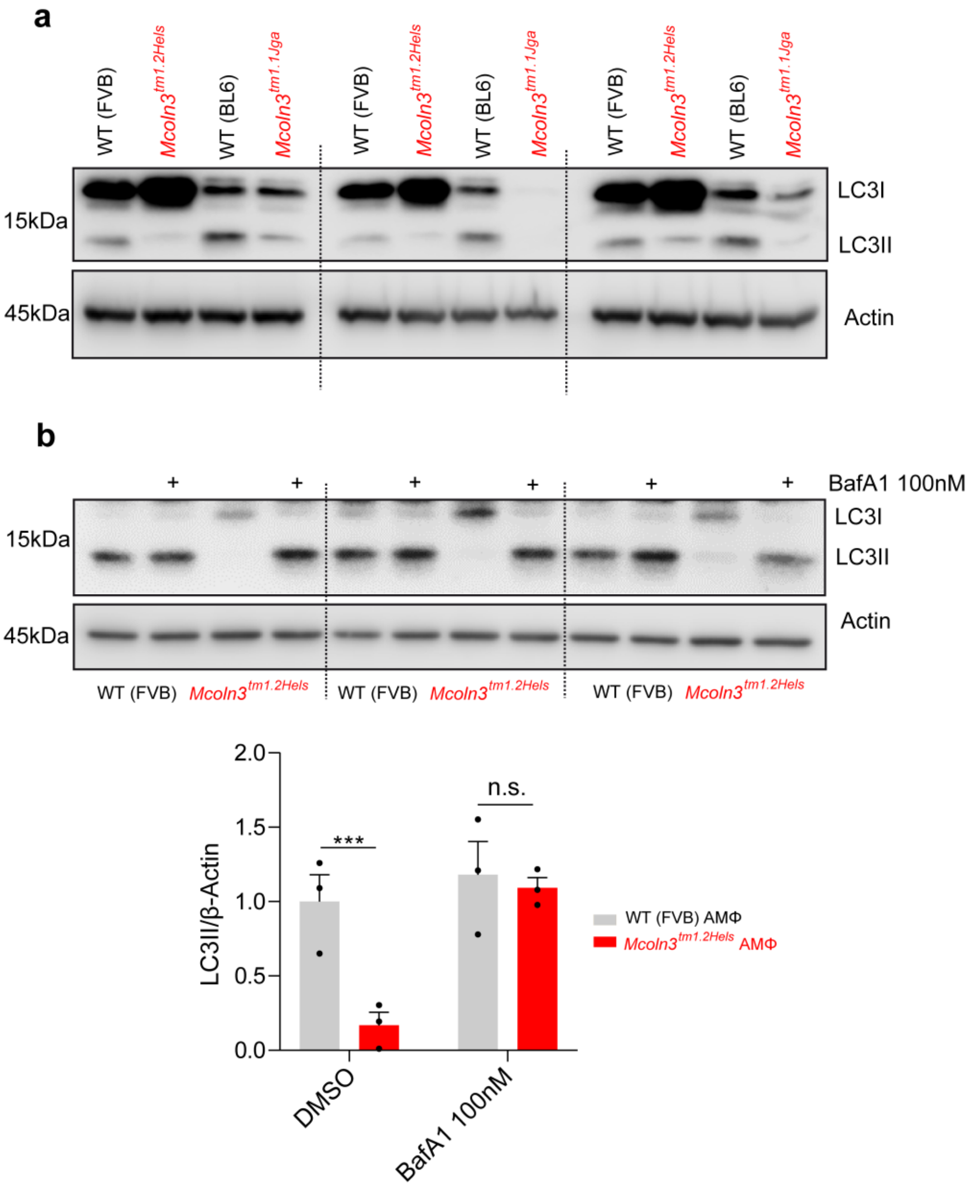


## Supplementary Figure S6

**LC3 expression analysis.** (**a**) Images of immunoblot analysis of endogenous LC3 (LC3I-II) in WT and *Trpml3*$^{-/-}$ AMΦ (*Mcoln3*$^{tm1.2Hels}$ and *Mcoln3*$^{tm1.1Jga}$). (**b**) Images of immunoblot analysis of endogenous LC3 (LC3I-II) in WT and *Trpml3*$^{-/-}$ AMΦ. Cells were treated for 5h with DMSO or bafilomycin (100nM). Plot shows the densitometry of LC3II band normalized to actin. Data are mean values ± SD, n = 3 lysates per condition pooled from n = 3 biologically independent experiments. P-values were calculated using two-tailed Student's t-test. *p-value < 0.05; **p-value < 0.01; ***p-value < 0.001. LC3 ab: NB100-2220 (1:1000); actin ab: Sc-47778 (1:1000); bafilomycin: B1793 (Merck). Source data are provided as a Source Data file.

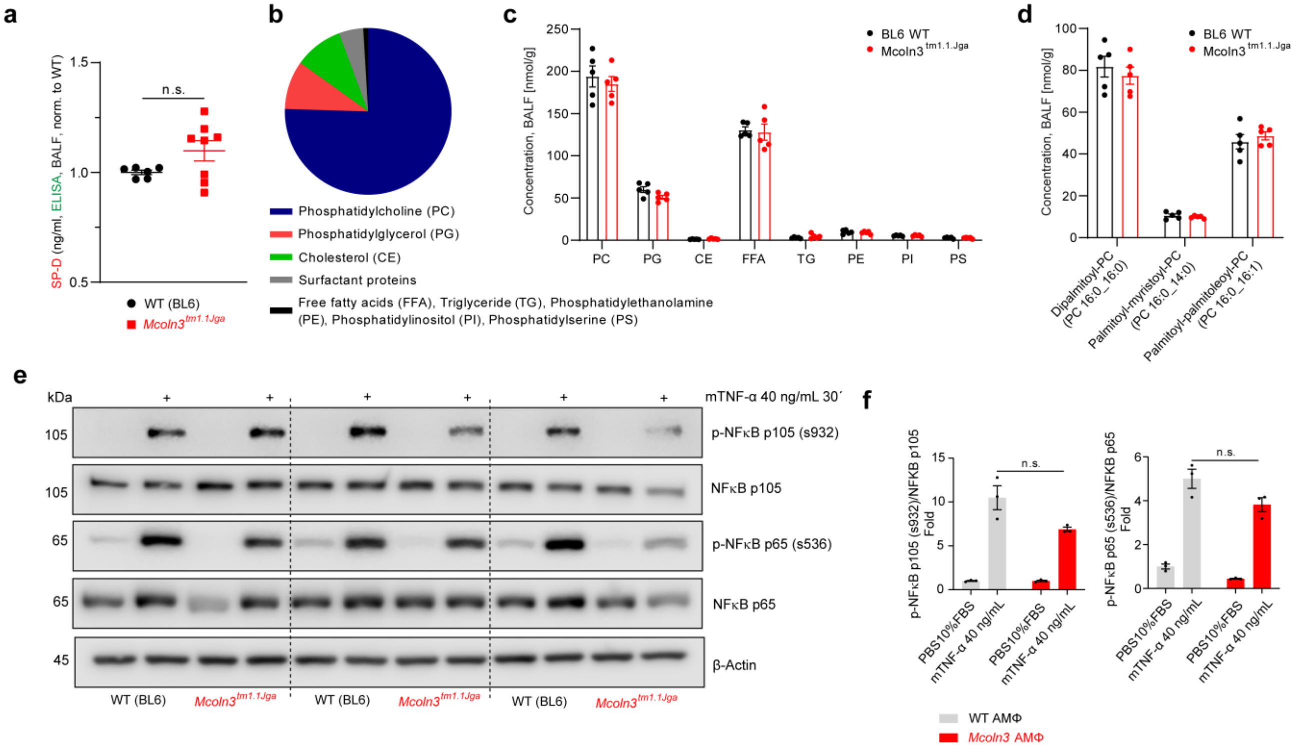


## Supplementary Figure S7

**Surfactant protein-D (SP-D) and lipidome analysis in BALF, and NF-κB expression analysis in AMΦ.** (**a**) ELISA data for SP-D using BALF isolated from WT and *Trpml3*$^{-/-}$ mice (*Mcoln3*$^{tm1.2Jga}$). Scatter plot shows single values and mean ± SEM. One single dot corresponds to BALF from one mouse. Unpaired, two-tailed Student's t-test applied. (**b**) Cartoon showing the composition of pulmonary surfactant according to literature. (**c-d**) Analysis of lipids (c) and of major phosphatidylcholine (PC) variants (d) in BALF samples isolated from WT and *Trpml3*$^{-/-}$ mice (*Mcoln3*$^{tm1.2Jga}$). One single dot corresponds to BALF from one mouse. Data are mean ± SEM. (**e**) Immunoblot analysis of the phosphorylation on serine s932 of NF-κB p105 and s536 of NF-κB p65 in WT and *Trpml3*$^{-/-}$ AMΦ (*Mcoln3*$^{tm1.1Jga}$). AMΦ were treated with 40 ng/mL of mTNFα for 30 min. (**f**) Bar plots are showing the densitometry of phospho NF-κB p105 (s932) on total NF-κB p105 and phospho NF-κB p65 (s536) on total NF-κB p65. Data are mean values ± SD, n = 3 lysates per condition pooled from n = 3 biologically independent experiments as shown in e. P-values calculated by two-tailed Student's t-test. *p-value < 0.05; **p-value < 0.01; ***p-value < 0.001. Reagents: Phospho-NF-κB p65 (Ser536) (93H1) Rabbit mAb 3033, NF-κB p65 (L8F6) Mouse mAb #6956, Phospho-NF-κB p105 (Ser932) (18E6) Rabbit mAb 4806, NF-κB1 p105/p50 (D4P4D) Rabbit mAb #13586, all used 1:1000. Mouse Tumor Necrosis Factor-α (mTNF-α) #5178 (40 ng/mL resuspended in PBS 10% FBS). Source data are provided as a Source Data file.

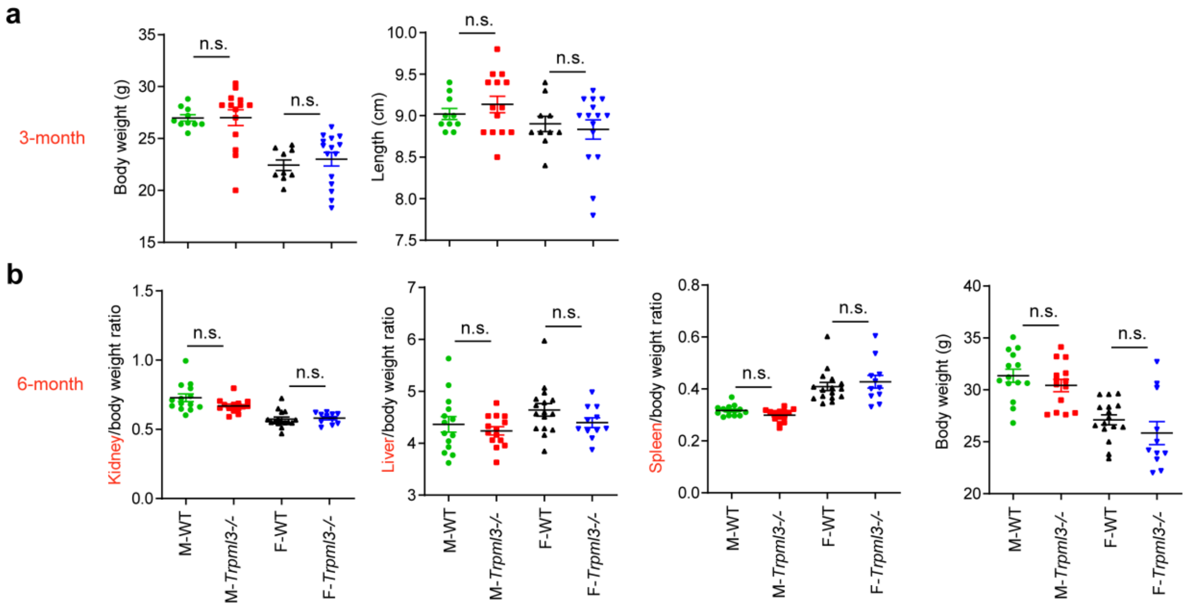


## Supplementary Figure S8

**Analysis of organ weight, body weight and length of 3- and 6-month old male and female WT and *Trpml3*$^{-/-}$ mice.** (**a-b**) Scatter plots showing single and average (mean ± SEM) organ to body weight ratios, body weight and length. Analysis was done using two-way ANOVA followed by Tukey's post-hoc test. Each dot represents one biologically independent sample. Source data are provided as a Source Data file.

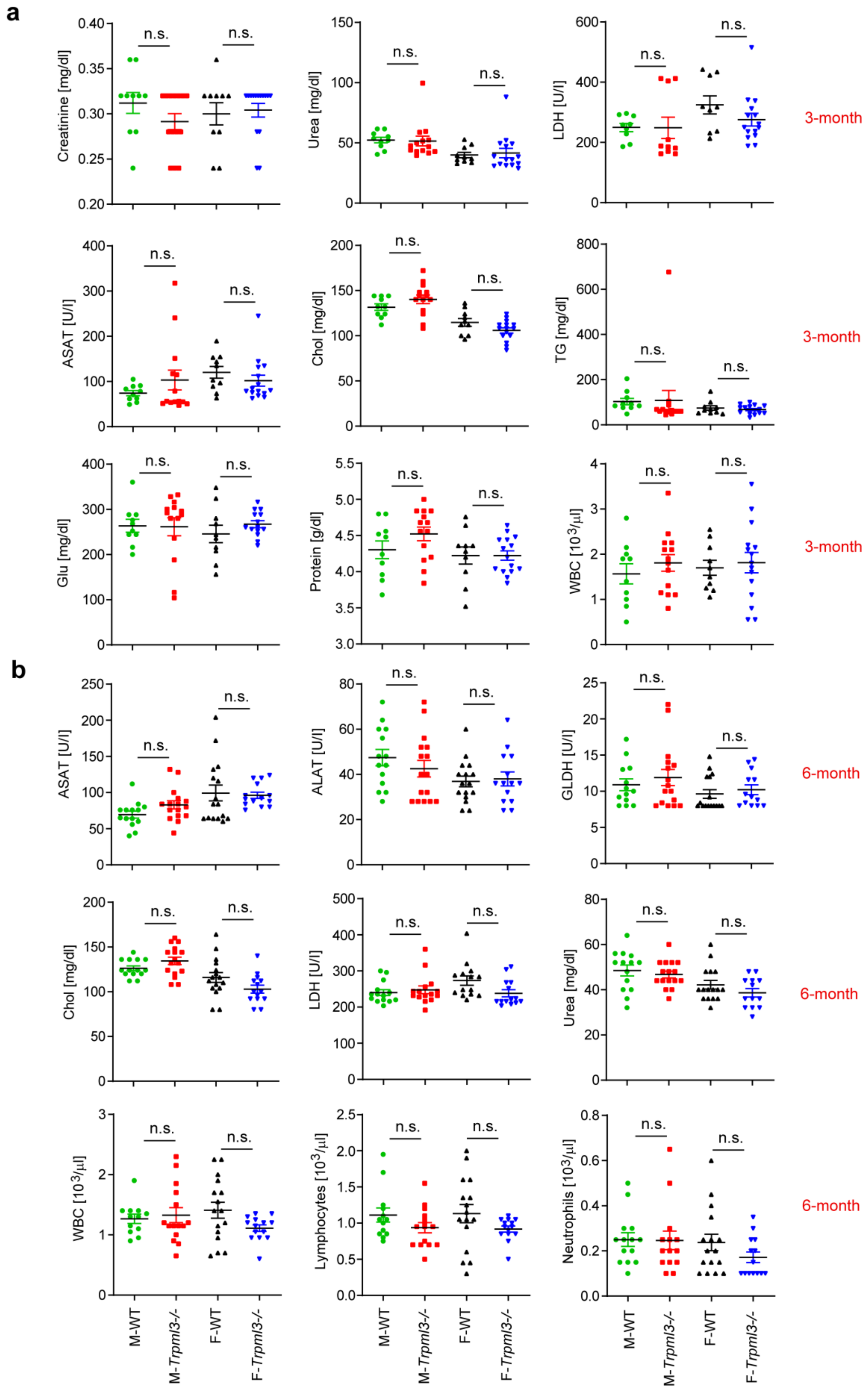
a
Creatinine [mg/dl]
Urea [mg/dl]
LDH [U/l]
3-month
ASAT [U/l]
Chol [mg/dl]
TG [mg/dl]
3-month
Glu [mg/dl]
Protein [g/dl]
WBC [10^3/µl]
3-month
n.s.
b
ASAT [U/l]
ALAT [U/l]
GLDH [U/l]
6-month
Chol [mg/dl]
LDH [U/l]
Urea [mg/dl]
6-month
WBC [10^3/µl]
Lymphocytes [10^3/µl]
Neutrophils [10^3/µl]
6-month
M-WT
M-Trpml3-/-
F-WT
F-Trpml3-/-

## Supplementary Figure S9

**Analysis of serum parameters of 3- and 6-month old male and female WT and *Trpml3*$^{-/-}$ mice.** (**a-b**) Scatter plots showing single and average (mean ± SEM) serum values of creatinine, urea, lactate dehydrogenase (LDH), aspartate amino transferrase (ASAT), alanine transaminase (ALAT), glutamate dehydrogenase (GLDH), cholesterol (Chol), triglycerides (TG), protein, glucose (Glu), white blood cells (WBC), lymphocytes, and neutrophils from male and female WT and *Trpml3*$^{-/-}$ mice. Analysis was done using two-way ANOVA followed by Tukey's post-hoc test. Each dot represents one biologically independent sample. Source data are provided as a Source Data file.

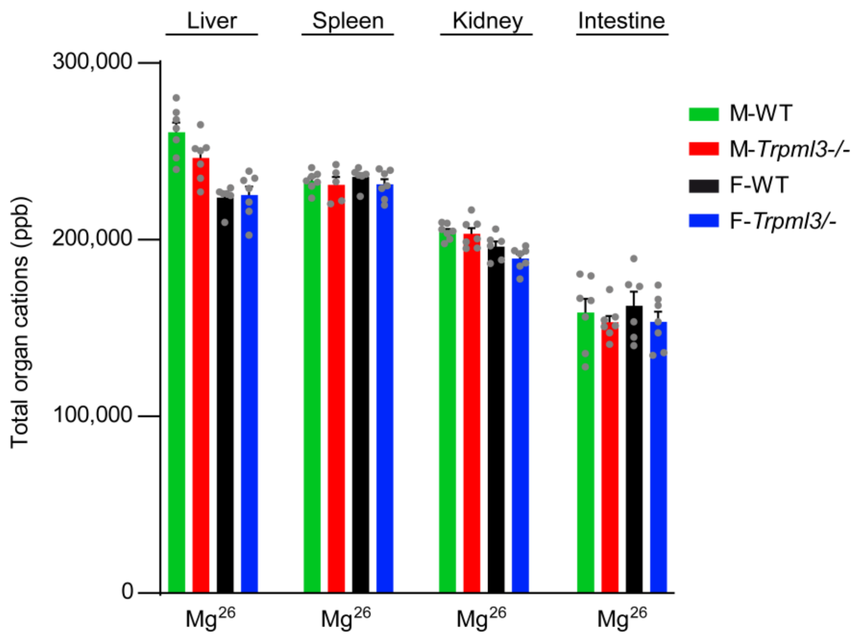


**Supplementary Figure S10**

**Total levels of magnesium in liver, spleen, kidney, and intestine of 6-month old male and female WT and *Trpml3*$^{-/-}$ mice.** Bar diagram displaying total organ magnesium (Mg26) levels (mean ± SEM) in 6-month old male and female WT and *Trpml3*$^{-/-}$ mice, measured using ICP-MS. Analysis was done using two-way ANOVA followed by Tukey's post-hoc test. No significant differences were found. Each dot represents one biologically independent sample. Source data are provided as a Source Data file.

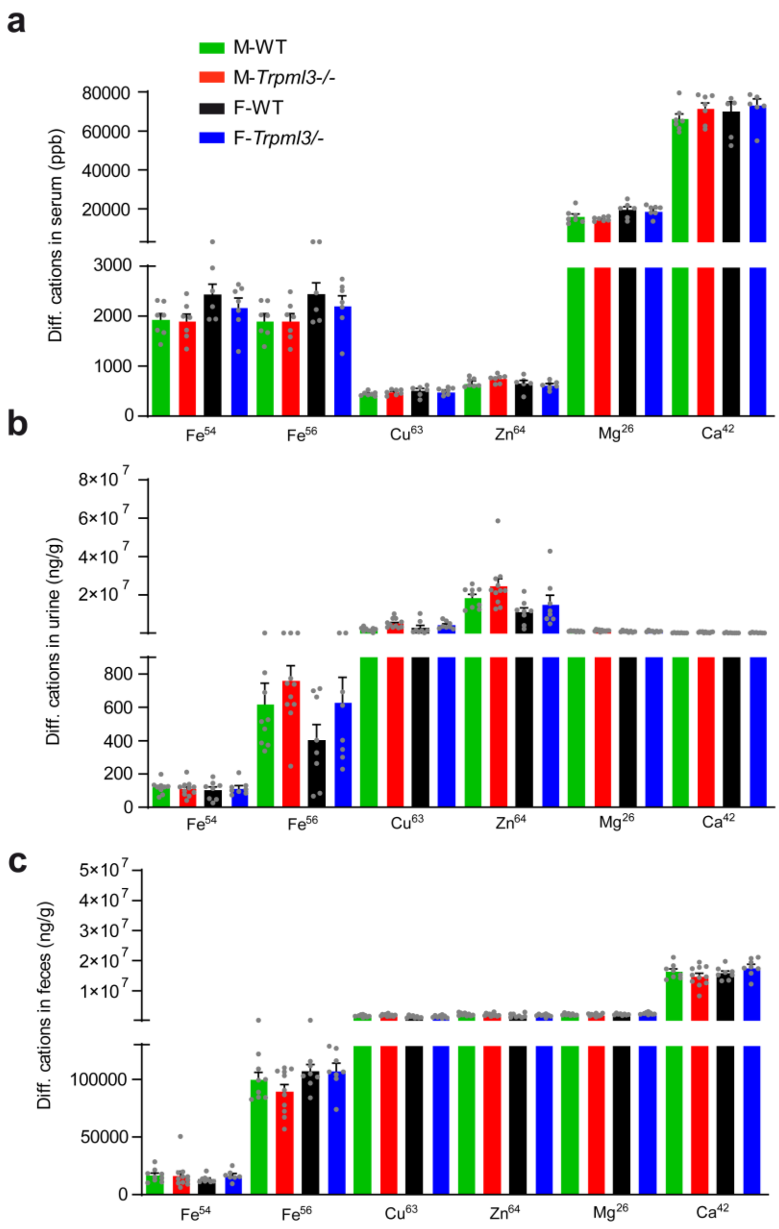


## Supplementary Figure S11

**Total levels of different elements/trace metals in serum, urine and feces of 6-month old male and female WT and *Trpml3*$^{-/-}$ mice.** (**a**) Bar diagram displaying average (mean ± SEM) serum iron (Fe54 and Fe56), magnesium (Mg26), calcium (Ca42), zinc (Zn64), and copper (Cu63) levels in 6-month old male and female WT and and *Trpml3*$^{-/-}$ mice, measured using ICP-MS. (**b-c**) Bar diagram displaying average (mean ± SEM) fecal (b) and urine (c serum iron (Fe54 and Fe56), magnesium (Mg26), calcium (Ca42), zinc (Zn64), and copper (Cu63) levels in 6-month old male and female WT and and *Trpml3*$^{-/-}$ mice, measured using ICP-MS. Analysis was done using two-way ANOVA followed by Tukey's post-hoc test. No significant differences were found. Each dot represents one biologically independent sample. Source data are provided as a Source Data file.

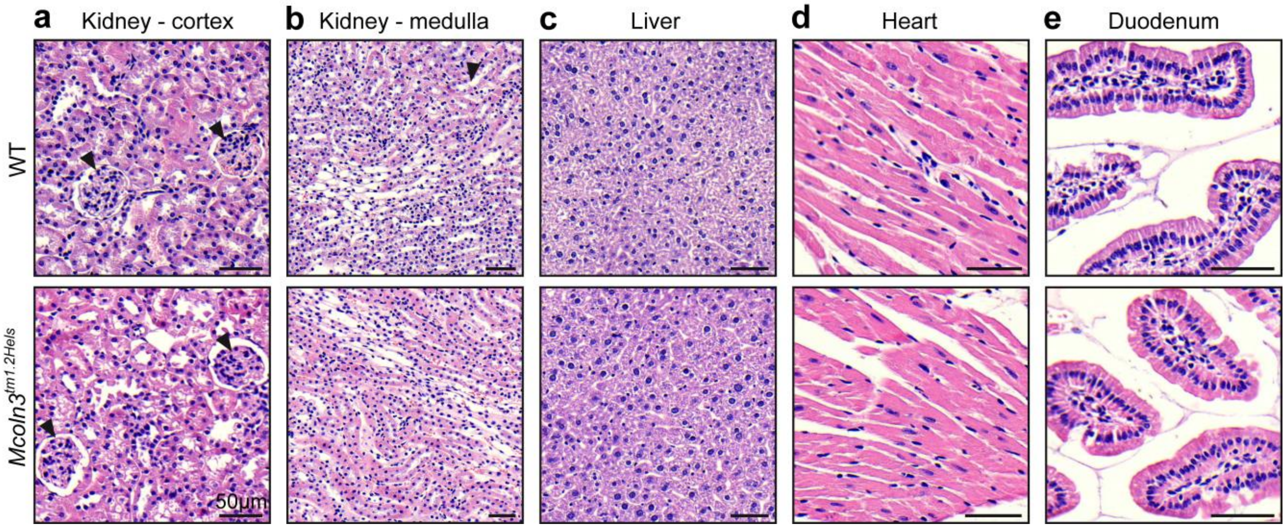


## Supplementary Figure S12

**Hematoxylin/eosin (HE) stainings of different organs from WT and *Trpml3*$^{-/-}$ mice.** Shown are HE stainings of formalin-fixed and paraffin-embedded 3 µm sections of several organs from WT and *Trpml3*$^{-/-}$ mice (*Mcoln3*$^{tm1.2Hels}$). Scale bars, 50 µm. (**a**) Image shows sagital section through the cortex of the kidney highlighting Bowman capsules (arrowheads). (**b**) Shown is an area of the medulla of the kidney containing several longitudinal collecting ducts. (**c**) Image of the liver revealing hepatocytes and sinusoids throughout the tissue. (**d**) Sections through the heart show striated muscle fibers formed by cardiomyocytes. (**e**) Image showing duodenal mucosa illustrating several villi. Experiments a-e were repeated 3 times with similar results.

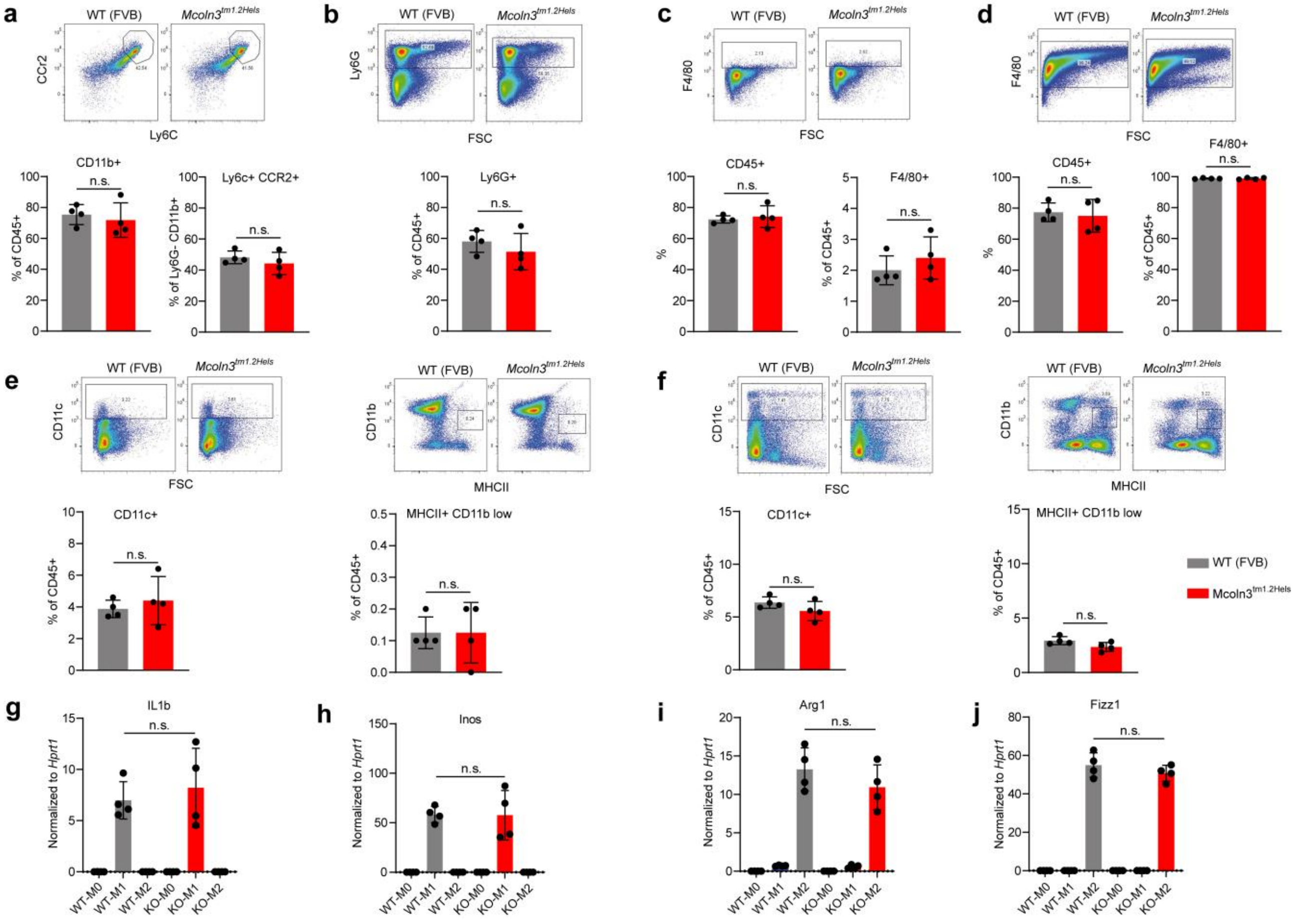


## Supplementary Figure S13

**Monocyte/macrophage development and macrophage polarization are similar in 5-month old WT and *Trpml3*$^{-/-}$ mice.** (**a-f**) Flow cytometry analysis and representative gating blots of monocytes in freshly isolated bone marrow (a, b, c, e), differentiated macrophages (d) and splenocytes (f) from wild-type (WT, n = 4) and *Trpml3*$^{-/-}$ (n=4) mice. a, Percentage of monocytes, Ly6c+ CCR2+ cells as a fraction of the CD11b+ population. b, Percentage of neutrophils, Ly6G+ cells as a fraction of the CD45+ population. c, Percentage of F4/80+ in freshly isolated bone marrow. d, Percentage of F4/80+ bone marrow derived macrophages (BMDM) differentiated 7 days in vitro. e, Percentage of dendritic cells, CD11c+ cells as a fraction of the CD45+ population or optional gating strategy MHCII$^{high}$;CD11b$^{low}$ cells as a fraction of the CD45+ population in freshly isolated bone marrow or f, in spleen. (**g-j**) 7 day differentiated bone marrow derived macrophages (BMDM) isolated from wild-type (WT, n = 4) and *Trpml3*$^{-/-}$ (n=4) mice were treated with 1 µg/ml LPS plus 20 ng/ml IFNγ (M1), 20 ng/ml IL-4 (M2) or medium alone (M0) for 24 h. mRNA expression levels of IL1b (g), Inos (h), Arg1(i) and Fizz1(j) by qPCR relative to WT M0 BMDMs. Data shown as mean ± SD. *P < 0.05, **P < 0.01, ***P < 0.001 and ****P < 0.0001, one-way ANOVA Tukey's multiple comparisons test. Source data are provided as a Source Data file.

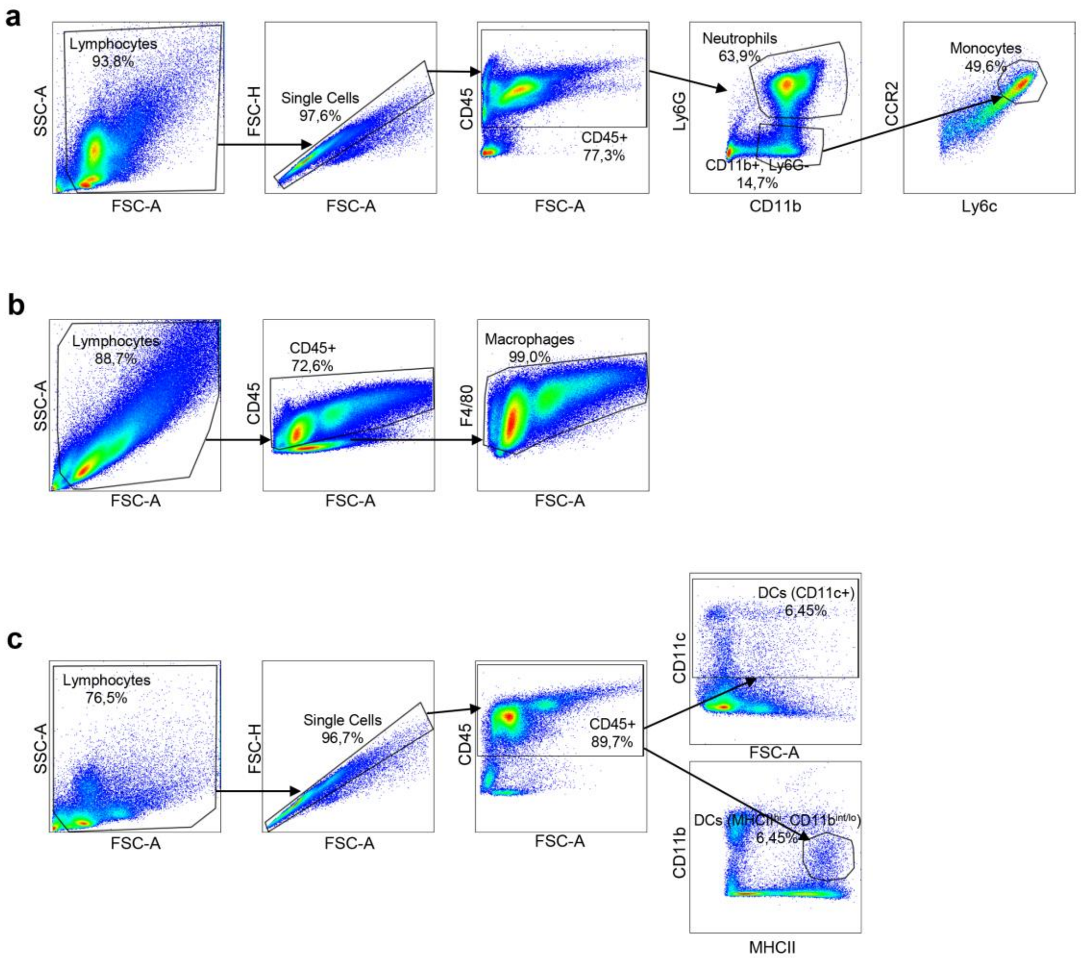


## Supplementary Figure S14

**Sequential gating strategies for identification of cell populations presented in Supplementary Figure S13a-e.** (**a**) Sequential and representative gating strategy applied to identify monocytes as Ly6C+ CCR2+ population (see S13a) and neutrophils as Ly6G+ cells (see S13b). FSC and SSC was used to gate lymphocytes and single cells. The CD45+ population was identified and further classified as neutrophils by using LY6G vs. FSC, as shown in S13b. To identify monocytes, the CD11b+;Ly6G- fraction was used and characterized by the markers Ly6C and CCR2. (**b**) Sequential and representative gating strategy applied to identify F4/80+ cells in freshly isolated bone marrow (see S13c) and in BMDM differentiated for 7 days in vitro (see S13d). FSC and SSC was used to gate lymphocytes and to identify CD45+ immune cells. In the following macrophages were gated as F4/80+ cells, here shown for BMDM differentiated for 7 days in vitro. (**c**) Sequential and representative gating strategy applied to identify dendritic cells as CD11c+ fraction or as MHCII$^{high}$;CD11b$^{low}$ population in freshly isolated bone marrow (see S13e) or in spleen (see S13f). FSC and SSC was used to gate lymphocytes and single cells. The CD45+ fraction was either characterized as CD11c+ dendritic cells or as MHCII$^{high}$;CD11b$^{low}$ dendritic cells.
