# Supplementary material for: Lung emphysema and impaired macrophage elastase clearance in mucolipin 3 deficient mice
Source: Nat Commun. 2022 Jan 14;13:318. doi: 10.1038/s41467-021-27860-x (PMC8760276; doi:10.1038/s41467-021-27860-x)
Supplement: Supplementary file 2 — Reporting Summary [file 41467_2021_27860_MOESM2_ESM.pdf]

nature research

Corresponding author(s): Christian Grimm
Ali Önder Yildirim
Martin Biel

Last updated by author(s): Nov 9, 2021

Reporting Summary

Nature Research wishes to improve the reproducibility of the work that we publish. This form provides structure for consistency and transparency in reporting. For further information on Nature Research policies, see our Editorial Policies and the Editorial Policy Checklist.

Statistics

For all statistical analyses, confirm that the following items are present in the figure legend, table legend, main text, or Methods section.

n/a	Confirmed	
☐	☒	The exact sample size (*n*) for each experimental group/condition, given as a discrete number and unit of measurement
☐	☒	A statement on whether measurements were taken from distinct samples or whether the same sample was measured repeatedly
☐	☒	The statistical test(s) used AND whether they are one- or two-sided<br>*Only common tests should be described solely by name; describe more complex techniques in the Methods section.*
☐	☒	A description of all covariates tested
☐	☒	A description of any assumptions or corrections, such as tests of normality and adjustment for multiple comparisons
☐	☒	A full description of the statistical parameters including central tendency (e.g. means) or other basic estimates (e.g. regression coefficient) AND variation (e.g. standard deviation) or associated estimates of uncertainty (e.g. confidence intervals)
☐	☒	For null hypothesis testing, the test statistic (e.g. *F*, *t*, *r*) with confidence intervals, effect sizes, degrees of freedom and *P* value noted<br>*Give P values as exact values whenever suitable.*
☒	☐	For Bayesian analysis, information on the choice of priors and Markov chain Monte Carlo settings
☒	☐	For hierarchical and complex designs, identification of the appropriate level for tests and full reporting of outcomes
☒	☐	Estimates of effect sizes (e.g. Cohen's *d*, Pearson's *r*), indicating how they were calculated

Our web collection on statistics for biologists contains articles on many of the points above.

Software and code

Policy information about availability of computer code

Data collection

Lung Function measurements were acquired using the flexiVent system running Flexiware v7.6.4 software (Scireq).
Histology images were aquired using Zeiss Mirax Microimmaging slide scanner running MIRAXDESK v1.12.25.1 and Mirax viewer v1.12.22.0 software (Zeiss, 3D Histech).
Immunofluorescence images from lung tissue from Trpml3-IRES-Cre/eR26-τGFP mice were aquired with ZEISS Axio Scan.Z1 Slide Scanner running ZEN software v2.0.0.0 (blue edition).
FACS data were acquired using BD FACSDiva software v8.0.1 ran on a BD LSRFortessa II or using BD FACSDiva software v6.1.3 ran on a BD FACSCanto II.
qPCR data was obtained using StepOne software v2.3 ran on a StepOnePlus Real-time PCR system (Applied Biosystems) or using LightCycler 480 software v1.5.1 ran on a Light Cycler 480 Instrument (Roche).
Calcium imaging was performed using a Polychrome V monochromator (TILL Photonics) running TILL Photonics Live Aquisition software v2.
Data from multiplex assay were collected using Bio-Plex System Luminex 100 (Bio-Rad) running Bio-Plex Manager software v4.1.1.
O.D. absorbances for ELISAs and Cytotoxicity assay were measured using FLUOstar Omega (BMG LABTECH) running Reader Control software v5.50 R4.
Automatic cell counting was performed using CASY 1 TT Cell Counter & Analyser System (Roche Innovatis).
Immunofluorescence images for Tf-trafficking assay, endocytosis experiment and LAMP1 translocation assay were aquired with a ZEISS LSM 880 running ZEN software v2.3 SP1.
Western blot bands were detected using the Odyssey FC Imaging System (LI-COR) running ImageStudio software v1.0.19.
For whole-EE, whole-RE, whole-LE/LY and whole cell patch clamp experiments currents were recorded using the patch-clamp amplifier HEKA EPC 10 running PatchMaster software v2x90.4.
For Hexosaminidase assay the fluorescence intensity at 450nm was measured using the SpectraMax iD3 (Molecular Devices) running SoftMax Pro Software v6.
Endolysosomal pH measurements were performed by fluorescence ratiometric imaging using an Olympus FV1000 confocal laser scanning and

images were aquired using a charge-coupled-device camera under control of MetaFluor software v7.6.5.0 (MDS Analytical Technolgies).
Mass spectrometric analysis of BALF was performed using a QTrap 5500 mass spectrometer (Sciex) with DMS, coupled to a Shimadzu Nexera X2 LC system, for flow injection, and the Lipidomics workflow manager software v.1.0.5.
Primers were designed using Primer-BLAST software (https://www.ncbi.nlm.nih.gov/tools/primer-blast/).

Data analysis	Histology data was analysed using the computer-assisted stereological toolbox software Visopharm Integrator System (VIS) v6.0.0.1765 (newCAST, Visiopharm).<br>The Seurat R package v3.2.2 was used for analysis of the RNA sequencing data.<br>Immunofluorescence images were processed using ZEN 2.6 (blue edition).<br>Immunofluorescence intensities were analysed using ImageJ (v1.52p).<br>Histology images were processed using CaseViewer 2.4 and Slide Converter 2.3.2 (3DHISTECH).<br>FACS data were analysed with FlowJo v10.0.7r2 software (FlowJo LLC, BD).<br>Western blot bands were quantified using ImageJ (v1.52p).<br>Data collected from Whole-cell patch-clamp experiments were analysed using the software IGOR Pro v6 (WaveMetrics).<br>Data collected from whole-EE, whole-RE and whole-LE/LY patch clamp experiments were analysed using the softwares OriginPro v6.1 and Origin v8 (OriginLab).<br>Microsoft Excel v 16.0.5017.1000 (part of Microsoft Office Professional Plus 2016) was used for normalisation and calculations of collected data.<br>Statistical analysis were conducted using GraphPad Prism v8.1.0 - v9.0.0.

For manuscripts utilizing custom algorithms or software that are central to the research but not yet described in published literature, software must be made available to editors and reviewers. We strongly encourage code deposition in a community repository (e.g. GitHub). See the Nature Research guidelines for submitting code & software for further information.

Data

Policy information about availability of data

All manuscripts must include a data availability statement. This statement should provide the following information, where applicable:
- Accession codes, unique identifiers, or web links for publicly available datasets
- A list of figures that have associated raw data
- A description of any restrictions on data availability

The scRNA-seq data used in this study are available in the Gene Expression Omnibus database under the accession codes
GSE124872 [https://www.ncbi.nlm.nih.gov/geo/query/acc.cgi?acc=GSE124872],
GSE151674 [https://www.ncbi.nlm.nih.gov/geo/query/acc.cgi?acc=GSE151674],
GSE185006 [https://www.ncbi.nlm.nih.gov/geo/query/acc.cgi?acc=GSE185006],
GSE27597 [https://www.ncbi.nlm.nih.gov/geo/query/acc.cgi?acc=GSE27597],
GSE8823 [https://www.ncbi.nlm.nih.gov/geo/query/acc.cgi?acc=GSE8823],
GSE2125 [https://www.ncbi.nlm.nih.gov/geo/query/acc.cgi?acc=GSE2125].
Source data are provided with this paper.

Field-specific reporting

Please select the one below that is the best fit for your research. If you are not sure, read the appropriate sections before making your selection.

☒ Life sciences ☐ Behavioural & social sciences ☐ Ecological, evolutionary & environmental sciences

For a reference copy of the document with all sections, see nature.com/documents/nr-reporting-summary-flat.pdf

Life sciences study design

All studies must disclose on these points even when the disclosure is negative.

Sample size	No statistical methods were used to predetermine sample size.<br>Sample sizes were chosen based on similar studies from the literature and were large enough to detect statistically significant differences between groups. Please see doi:10.1038/s41586-020-2882-8 and doi:10.1165/rcmb.2008-0487OC and doi:10.18632/oncotarget.4027.<br>Exact sample sizes are indicated in the figure legends.
Data exclusions	Significant outliers identified by using Grubbs` test (see doi:10.1214/aoms/1177729885 and doi:10.2307/1266761) were excluded, significance level: p=0,05. This applied to the following experiments:<br>FACS analysis of BAL (as shown in Fig. 1g): one sample needed to be excluded due to mistakes during sample processing<br>MMP-12 ELISA of BALF (as shown in Fig. 4c): one extremeley high outlier for each group, respectively, was identified and excluded<br>Counts of E-fibers/field (as shown in Fig. 4n): one outlier was identified and excluded from the group "PBS, BL6 WT"<br>TIMP-2 ELISA of BALF (as shown in Fig. 8b): one outlier was identified and excluded from the group "WT (BL6)"<br>MCL measurements (as shown in Fig. 9b): one outlier was identified and excluded from the group "Mcoln3tm1.1Jga, CS"
Replication	All findings were reproducible with group sizes and number of independent repeats as mentioned in the figure legends or methods.
Randomization	For quantification of airspace enlargements the computer-assisted stereological toolbox software Visopharm Integrator System randomly

chose 30 fields of view per lung/replica to be analysed.
For lung function measurements grouping of mice was not performed randomly to ensure that the mice within groups have the same gender, age and correct genotype.
For all other experiments based on cells or samples isolated from mice, WT and TRPML3 KO mice were processed in parallel and mice were specifically chosen to match age and gender between the genotypes.
For all in vitro cell culture experiments wells were randomly allocated to experimental groups.

Blinding

Blinding was not possible, because most of the experiments were planned and performed by the same person. This way, it was ensured that each experiment contained all groups and appropiate controls.

Reporting for specific materials, systems and methods

We require information from authors about some types of materials, experimental systems and methods used in many studies. Here, indicate whether each material, system or method listed is relevant to your study. If you are not sure if a list item applies to your research, read the appropriate section before selecting a response.

Materials & experimental systems

n/a	Involved in the study	
☐	☒	Antibodies
☐	☒	Eukaryotic cell lines
☒	☐	Palaeontology and archaeology
☐	☒	Animals and other organisms
☒	☐	Human research participants
☒	☐	Clinical data
☒	☐	Dual use research of concern

Methods

n/a	Involved in the study	
☒	☐	ChIP-seq
☐	☒	Flow cytometry
☒	☐	MRI-based neuroimaging

Antibodies

Antibodies used	Immunofluorescence:<br>anti-mouse F4/80, clone Cl:A3-1 (MCA497G, AbD Serootec)<br>anti-mouse CD45R, clone RA3-6B2 (550286, BD Biosciences)<br>anti-mouse CD3, polyclonal (C7930, Sigma)<br>anti-mouse SFTPC, polyclonal (AP13684b, Abcepta)<br>anti-mouse CD8α, clone EPR21769 (217344, Abcam)<br>anit-rabbit-Cy5, 1:500 (711-175-152, Jackson ImmunoResearch)<br>anti rat-Cy3 (12-165-153, Jackson ImmunoResearch)<br>anti-mouse EEA1, clone C45B10 (3288, Cell signaling)<br>anti-mouse LAMP1, clone 1D4B (9992, SantaCruz)<br>anti-rat-AlexaFluor488 (A11006, ThermoFisher)<br><br>Fluorescence-activated cell sorting (FACS):<br>TruStain FcX (anti-mouse CD16/32), clone 93 (101319, Biolegend)<br>anti-mouse CD24-PerCP/Cy5.5, clone M1/69 (101823, BioLegend)<br>anti-mouse CD64-APC, clone X54-5/7.1 (139305, BioLegend)<br>anti-mouse CD45R/B220-AlexaFluor700, cloneRA3-6B2 (103231, BioLegend)<br>anti-mouse CD45-PacBlue, clone 30-F11 (103125, BioLegend)<br>anti-mouse Ly-6G-PE/Dazzle™594, clone 1A8 (127647, BioLegend)<br>anti-mouse CD11c-BV605, clone N418 (117333, BioLegend)<br>anti-mouse CD11b-BV785, clone M1/70 (101243, BioLegend)<br>anti-mouse CD3-PE/Cy7, clone 17A2 (100219, BioLegend)<br>anti-mouse MHCII-PE, clone NIMR-4 (1895-09, SouthernBiotech)<br>anti-mouse CD16/CD32, clone 93 (14-0161-82, ThermoFisher)<br>anti-mouse CD45, clone 30F11 (130-123-900 , Miltenyi Biotec)<br>anti-mouse F4/80, clone REA126 (130-118-466, Miltenyi Biotec)<br>anti-mouse CD11b, clone M1/70.15.11.5 (130-091-240, Miltenyi Biotec)<br>anti-mouse CD11c, clone N418 (130-119-802, Miltenyi Biotec)<br><br>Western Blot:<br>anti-mouse TfR, clone H68.4 (13-6800, ThermoFisher)<br>anti-mouse LC3B, polyclonal (100-2220,Novus Biologicals)<br>anti-mouse Phospho-NFKB p65 (Ser536), clone 93H1 (3033, Cell Signaling)<br>anti-mouse NFKB p65, clone L8F6 (6956, Cell Signaling)<br>anti-mouse Phospho-NFKB p105 (Ser932), clone 18E6 (4806, Cell Signaling)<br>anti-mouse NFKB1 p105/p50, clone D4P4D (13586, Cell Signaling)<br>anti-mouse ß-actin, clone 13E5 (4970, Cell Signaling)<br>anti-mouse ß-actin, clone C4 (47778, Santa Cruz)
Validation	Only well established and commercially avaible antibodies were used.<br>Validation of commercial antibodies was carried out by the manufacturer performing regular quality control of each lot as stated in the data sheet or on the manufacturers website.<br>E.g. Biolegend: "Each lot of this antibody is quality control tested by immunofluorescent staining with flow cytometric analysis."<br>E.g. Cell Signaling: "When an antibody is recommend for a particular application, it indicates that the antibody has passed rigorous application-specific testing standards."<br>E.g. ThermoFisher: Antibodies undergo a "rigorous 2-part testing approach" including "Target specifity verification" and "Functional application validation".<br>E.g. Miltenyi Biotec: Antibodies are tested for "Lot-to-lot consistent performance". Antibody specifity is validated using "Knockout validation via target genome editing". Antibody sensitivity is evaluated through "Functional testing of every product prior to release".<br>E.g. Merck (Sigma): "Each of the thousands of antibodies in our portfolio are certified through our standard validation process to ensure quality and reproducibility."<br>E.g. Abcam: "To confirm antibody specificity, we have introduced knockout validation as a standard level of validation, with over 800 knockout-validated antibodies and counting."<br>All antibodies were used for applications that were tested by the manufacturer, as specified on the respective data sheet. We validated dilutions of antibodies based on recommendations of the manufacturer or on their use in previous publications.

Eukaryotic cell lines

Policy information about cell lines

Cell line source(s)	HEK-293, #CRL-1573, ATCC
Authentication	The cell line was not authenticated.
Mycoplasma contamination	The cell line was routinely tested, and tested negative for Mycoplasma.

Commonly misidentified lines (See ICLAC register)	No commonly misidentified cell lines were used in the study.

Animals and other organisms

Policy information about studies involving animals; ARRIVE guidelines recommended for reporting animal research

Laboratory animals	The following mouse models were used:<br>Mcoln3tm1.2Hels (genetic background: FVB)<br>Mcoln3tm1.1Jga (genetic background: C57BL/6)<br>Mcoln1tm1Sasl (genetic background: C57BL/6)<br>Trpml3IRES-Cre/eR26-tGFP (mixed genetic background: C57BL6/129Sv)<br>Female and male mice aged 2-6 months were used. For further details see figure legends or methods.
Wild animals	No wild animals were used in the study.
Field-collected samples	No field-collected samples were used in the study.
Ethics oversight	All research performed complies with all relevant ethical regulations. Animals were used under animal protocols approved by the government (Regierung von Oberbayern, ROB-55.2-2532.Vet_02-17-170 and ROB-55.2-2532.Vet_02-18-6), and University of Munich (LMU) and the German Center for Lung Research (DZL) Institutional Animal Care Guidelines.

Note that full information on the approval of the study protocol must also be provided in the manuscript.

Flow Cytometry

Plots

Confirm that:

- [x] The axis labels state the marker and fluorochrome used (e.g. CD4-FITC).
- [x] The axis scales are clearly visible. Include numbers along axes only for bottom left plot of group (a 'group' is an analysis of identical markers).
- [x] All plots are contour plots with outliers or pseudocolor plots.
- [x] A numerical value for number of cells or percentage (with statistics) is provided.

Methodology

Sample preparation	BALF was isolated from Trpml3-IRES-Cre/eR26-τGFP mice. Then lungs were perfused with 20 ml ice-cold PBS, removed and placed on a petri dishes with PBS. Lung tissue was minced into pieces using scalpels and processed in digestion buffer containing collagenase and DNAse (30 min at 37°C). Homogenized lungs were passed through nylon strainers (100 µm and 30 µm) to obtain a single-cell-suspension. Remaining erythrocytes were lysed and resultant cells were incubated with Fc blocking antibody, stained with viability dye and a mixture of fluorochrome-conjugated antibodies for 20 min at 4°C (for list of antibodies, see above). After incubation cells were washed and analysed.<br><br>Bone marrow was flushed from femurs and tibias of Trpml3-/- mice and WT littermate controls with RPMI-1640 medium. Suspension was passed through 40µm filters, counted and resuspended in RPMI-1640 medium supplemented with 5% fetal bovine serum, 50µM β-mercaptoethanol and 100 U/ml penicillin and streptomycin. 2x10^6 cells/ml were plated in 24 well plates and 20ng/mL of murine recombinant M-CSF were added to the medium. Cells were maintained at 37°C, 5% CO2 for 7days changing medium every 3rd day and carefully discarding non-adherent cells. On day 7, fresh medium without M-CSF was added and left overnight. FACS analysis were performed on freshly harvested bone marrow as well as day 7 bone marrow derived macrophages.
Instrument	BD LSRFortessa II<br>BD FACSCanto II
Software	BD FACSDiva software v8.0.1<br>BD FACSDiva software v6.1.3
Cell population abundance	Samples were not sorted for post-sort fractions.
Gating strategy	Lung tissue:<br>FSC and SSC were used to identify lymphocytes and exclude doublets or debris. After gating for live immune cells (LD-, CD45 +) only TRPML3+ cells (GFP+) were selected. In the following steps various immune cell types were excluded: T-cells (CD3e+, B220-), B-cells (CD3e-, B220+), neutrophils (Ly6G). After excluding small subsets of CD11b-/CD11c- and MHCII- cells, a big population of MΦ (CD64+ and CD24-) and a very small one of DC (CD64- and CD24+) were identified. The MHCII- subset provided monocytes/undifferentiated macrophages (CD11b+ and CD64-) and NK-cells (CD11blow and CD64-). DC were further classified into CD11b+DC, CD103+DC (CD11b-), and eosinophils (CD24+ and CD11b+) were identified. The population of MΦ was divided into CD11b+ interstitial macrophages (IMΦ) and CD11b- alveolar macrophages (AMΦ).<br><br>BALF:

Similar gating strategy was used as described for lung tissue.

Cells from Bone marrow:
Monocytes were gated as Ly6c+ CCR2+ fraction of CD11b+ population.
Neutrophils were gated as Ly6G+ fraction of CD11b+ population.
Macrophages were identified using F4/80 and FSC. Macrophages constitue the F4/80+ population.
Dendritic cells were identified as CD11c+ population using CD11c and FSC. Alternatively, dendritic cells were gated using MHCII vs. CD11b and identified as MHCII+ CD11b low population.

☒ Tick this box to confirm that a figure exemplifying the gating strategy is provided in the Supplementary Information.
